# Gradient projection method with a new step size for the split feasibility problem

**DOI:** 10.1186/s13660-018-1712-0

**Published:** 2018-05-18

**Authors:** Pattanapong Tianchai

**Affiliations:** Faculty of Science, Maejo University, Chiangmai, Thailand

**Keywords:** 47H05, 47H09, 47H20, Split feasibility problem, Split common fixed point problem, Gradient projection method

## Abstract

In this paper, we introduce an iterative scheme using the gradient projection method with a new step size, which is not depend on the related matrix inverses and the largest eigenvalue (or the spectral radius of the self-adjoint operator) of the related matrix, based on Moudafi’s viscosity approximation method for solving the *split feasibility problem* (SFP), which is to find a point in a given closed convex subset of a real Hilbert space such that its image under a bounded linear operator belongs to a given closed convex subset of another real Hilbert space. We suggest and analyze this iterative scheme under some appropriate conditions imposed on the parameters such that another strong convergence theorems for the SFP are obtained. The results presented in this paper improve and extend the main results of Tian and Zhang (J. Inequal. Appl. 2017:Article ID 13, 2017), and Tang et al. (Acta Math. Sci. 36B(2):602–613, 2016) (in a single-step regularized method) with a new step size, and many others. The examples of the proposed SFP are also shown through numerical results.

## Introduction

Throughout this paper, we always assume that *C* and *Q* be closed convex subsets of two real Hilbert spaces *H* and *K*, respectively with inner product and norm are denoted by $\langle\cdot,\cdot \rangle$ and $\|\cdot\|$, respectively. Let $A:H \rightarrow K$ be a bounded linear operator. The *split feasibility problem* (SFP) which was first introduced by Censor and Elfving [3] is to find

1.1 $$ x^{*} \in C \quad\text{such that}\quad Ax^{*} \in Q. $$

Suppose that $P_{C}$ and $P_{Q}$ are the orthogonal projections onto the sets *C* and *Q*, respectively. Assume that SFP (1.1) is consistent. We observe that $x^{*} \in C$ solves the SFP (1.1) if and only if it solves the fixed point equation

$$x^{*} = P_{C} \bigl(I-\gamma A^{*}(I-P_{Q})A \bigr)x^{*}, $$

where $\gamma> 0$ is any positive constant, *I* is the identity operator on *H* or *K*, and $A^{*}$ denotes the adjoint of *A*. To solve SFP (1.1) in the setting of a finite-dimensional real Hilbert space case, Byrne [4] proposed the so-called *CQ* algorithm based on the Picard iteration method as follows:

1.2 $$ x_{n+1} = P_{C} \bigl(I-\gamma A^{t} (I-P_{Q})A \bigr)x_{n},\quad \forall n=0,1,2,\ldots, $$

where $\gamma\in(0,\frac{2}{L})$ such that *L* being the largest eigenvalue of the matrix $A^{t} A$ as $A^{t}$ stands for matrix transposition of *A*. He proved that the sequence $\{x_{n}\}$ generated by (1.2) converges strongly to the SFP (1.1).

In [5], Yang presented a relaxed *CQ* algorithm for solving the SFP (1.1), where he used two halfspaces $C_{n}$ and $Q_{n}$ in place of *C* and *Q*, respectively, and at the *n*th iteration, the orthogonal projections onto $C_{n}$ and $Q_{n}$ are easily executed.

Both the *CQ* algorithm and the relaxed *CQ* algorithm used a fixed step size related to the largest eigenvalue of the matrix $A^{*} A$ or the spectral radius of the self-adjoint operator $A^{*}A$, which sometimes affects convergence of the algorithms. In [6], Qu and Xiu presented a modification of the *CQ* algorithm and the relaxed *CQ* algorithm by adopting the Armijo-like searches, which need not to compute the matrix inverses and the largest eigenvalue of the matrix $A^{*} A$. The *CQ*-like algorithms are also proposed subsequently [3, 7–10].

In all these *CQ*-like algorithms for the SFP (1.1), in order to get the step size, one has to compute the largest eigenvalue of the related matrix or use some line search scheme which usually requires many inner iterations to search for a suitable step size in every iteration.

We note that a point *x* solves the SFP (1.1) means that there is an element $x \in C$ such that $Ax-x^{*} = 0$ for some $x^{*}\in Q$. This motivates us to consider the distance function $d(Ax,x^{*}) = \| Ax-x^{*}\|$ for all $x\in C$, and the constrained convex minimization problem:

$$\min_{x\in C,x^{*}\in Q} \frac{1}{2} \bigl\Vert Ax - x^{*} \bigr\Vert ^{2} $$

such that minimizing with respect to $x^{*} \in Q$. Let $g:C \rightarrow \mathbb{R}$ be a continuous differentiable function. First makes us consider the minimization:

1.3 $$ \min_{x\in C} g(x) := \frac{1}{2} \Vert Ax - P_{Q} Ax \Vert ^{2} $$

is ill-posed. Therefore, Xu [11] considered the following Tikhonov regularization problem:

$$\min_{x\in C} g_{\epsilon}(x) := g(x)+\frac{\epsilon}{2} \Vert x \Vert ^{2} = \frac{1}{2} \Vert Ax-P_{Q} Ax \Vert ^{2}+ \frac{\epsilon}{2} \Vert x \Vert ^{2}, $$

where $\epsilon> 0$ is the regularization parameter. We know that the gradient $\nabla g_{\epsilon}$ of $g_{\epsilon}$ as

$$\nabla g_{\epsilon}(x) = \nabla g(x) + \epsilon I = A^{*}(I-P_{Q})A+ \epsilon I,\quad \forall x\in C, $$

such that $\nabla g_{\epsilon}(x)$ is $(\epsilon+\|A\|^{2})$-Lipschitzian continuous (that is, $\|\nabla g_{\epsilon}(x)-\nabla g_{\epsilon}(y)\| \leq (\epsilon+\|A\|^{2})\|x-y\| $ for all $x,y\in C$) and *ϵ*-strongly monotone (that is, $\langle\nabla g_{\epsilon}(x)-\nabla g_{\epsilon}(y) , x-y \rangle\geq\epsilon\|x-y\|^{2}$ for all $x,y \in C$).

Assume that the constrained convex minimization problem (1.3) is consistent. In [11], Xu suggested a single-step regularized method based on the Picard iteration method in the setting of an infinite-dimensional real Hilbert space as follows:

1.4 $$ \begin{aligned}[b]x_{n+1} &= P_{C}(I-\gamma_{n} \nabla g_{\epsilon_{n}})x_{n} \\ &= P_{C} \bigl(I-\gamma_{n} \bigl(A^{*}(I-P_{Q})A+ \epsilon_{n} I \bigr) \bigr)x_{n}, \quad\forall n=0,1,2,\ldots.\end{aligned} $$

He proved that the sequence $\{x_{n}\}$ generated by (1.4) converges in norm to the minimum-norm solution (1.3) of the SFP (1.1), provided the parameters $\{\epsilon_{n}\},\{\gamma _{n}\} \subset(0,1)$ satisfy the following conditions:

(i) $\lim_{n\rightarrow\infty}\epsilon_{n} = 0$ and $0< \gamma_{n} \leq\frac{\epsilon_{n}}{\|A\|^{2}+\epsilon_{n}}$,
(ii) $\sum_{n=0}^{\infty}\epsilon_{n} \gamma_{n} = \infty$,
(iii) $\lim_{n\rightarrow\infty}\frac{|\gamma_{n+1}-\gamma _{n}|+\gamma_{n} |\epsilon_{n+1}-\epsilon_{n} |}{(\epsilon_{n+1} \gamma _{n+1})^{2}} = 0$.

In [1], Tian and Zhang suggested a single-step regularized method based on the Picard iteration method in the setting of an infinite-dimensional real Hilbert space as follows:

1.5 $$ \begin{aligned}[b]x_{n+1} &= P_{C}(I-\lambda\nabla g_{\epsilon_{n}})x_{n} \\ &= P_{C} \bigl(I-\lambda \bigl(A^{*}(I-P_{Q})A+ \epsilon_{n} I \bigr) \bigr)x_{n}, \quad\forall n=0,1,2,\ldots.\end{aligned} $$

They proved that the sequence $\{x_{n}\}$ generated by (1.5) converges in norm to the minimum-norm solution (1.3) of the SFP (1.1), provided the parameters $\{\epsilon_{n}\} \subset (0,1)$ and *λ* satisfy the following conditions:

(i) $0< \lambda< \frac{2}{\|A\|^{2}+2}$,
(ii) $\lim_{n\rightarrow\infty}\epsilon_{n} =0$ and $\sum_{n=0}^{\infty}\epsilon_{n} = \infty$,
(iii) $\sum_{n=0}^{\infty}|\epsilon_{n+1}-\epsilon_{n} | < \infty$.

We observe that in the proof of their proposed results, $\sum_{n=0}^{\infty}|\epsilon_{n+1}-\epsilon_{n} | < \infty$ of the control condition (iii) can be removed using the NST-condition (II) [12] (see also [13, 14]).

The SFP (1.1) is an important and has been widely studied because it plays a prominent role in the signal processing and image reconstruction problem. Initiated by the SFP, several split type problems have been investigated and studied, for examples, the split variational inequality problem (SVIP) (see [15]) and the split common null point problem (SCNP). We will consolidate these problems. Let $S:H\rightarrow H$ and $T:K\rightarrow K$ be two operators with nonempty fixed point sets $\operatorname{Fix}(S):=\{x\in H : x=S(x)\}$ and $\operatorname{Fix}(T)$, respectively. If *S* be a nonexpansive mapping (that is, $\|Sx-Sy\| \leq\|x-y\|$ for all $x,y \in H$), then $\operatorname{Fix}(S)$ is closed and convex (see [16]). The *split common fixed point problem* (SCFP) is to find

1.6 $$ x^{*} \in\operatorname{Fix}(S) \quad\text{such that}\quad Ax^{*} \in\operatorname{Fix}(T). $$

If $S=P_{C}$ and $T=P_{Q}$ then $\operatorname{Fix}(S) = C$ and $\operatorname{Fix}(T) = Q$, and hence the SCFP (1.6) immediately reduces to the SFP (1.1).

Assume that the SCFP (1.6) is consistent. In [17], Censor and Segal proposed and proved a strong convergence theorem for the SCFP (1.6) based on the Picard iteration method, in the case that the directed operators *S* (that is, $\langle x-Sx,Sx-y \rangle\geq0$ for all $y \in\operatorname {Fix}(S)$ and $x\in H$, for instance $S=P_{C}$) and *T*, still in a finite-dimensional real Hilbert space to extend the iteration method (1.2) of Byrne as follows:

$$x_{n+1} = S \bigl(I-\gamma A^{t} (I-T)A \bigr)x_{n},\quad \forall n=0,1,2,\ldots, $$

where $\gamma\in(0,\frac{2}{L})$.

In [18], Kraikaew and Saejung proposed and proved a strong convergence theorem for the SCFP (1.6) based on the Halpern iteration method, in the case that the quasi-nonexpansive mappings *S* (that is, $\|Sx-p\| \leq\|x-p\|$ for all $x\in H$ and $p\in\operatorname{Fix}(S)$) and *T* such that both $I-S$ and $I-T$ are demiclosed at zero on an infinite-dimensional real Hilbert space as follows:

$$x_{n+1} = \alpha_{n} x_{0} + (1- \alpha_{n}) S \bigl(I-\gamma A^{*} (I-T)A \bigr)x_{n},\quad \forall n=0,1,2,\ldots, $$

where $\gamma\in(0,\frac{1}{L})$, $\{\alpha_{n}\} \subset(0,1)$, $\lim_{n\rightarrow\infty} \alpha_{n} =0$ and $\sum_{n=0}^{\infty}\alpha_{n} = \infty$.

In [2], Tang et al. proposed and proved a strong convergence theorem for the SCFP (1.6) based on the viscosity approximation method, in the case that the firmly nonexpansive mappings *S* (that is, $\|Sx-Sy\|^{2} \leq\|x-y\|^{2}-\|(I-S)x-(I-S)y\|^{2}$ for all $x,y\in H$) and *T* such that both $I-S$ and $I-T$ are demiclosed at zero, and an *α*-contraction mapping $h:H \rightarrow H$ with $\alpha\in(0,1)$ (that is, $\|h(x)-h(y)\| \leq\alpha\|x-y\|$ for all $x,y \in H$) on an infinite-dimensional real Hilbert space in a single-step regularized method as follows:

$$x_{n+1} = \alpha_{n} x_{n} + \beta_{n} h(x_{n}) +\gamma_{n} S \bigl(I-\xi_{n} A^{*} (I-T)A \bigr)x_{n}, \quad\forall n=0,1,2,\ldots, $$

where $\alpha_{n}+\beta_{n}+\gamma_{n} = 1$, $\xi_{n} = \frac{\rho_{n} \| (I-T)Ax_{n}\|^{2}}{2\| A^{*}(I-T)Ax_{n} \|^{2}}$, $\{\rho_{n}\} \subset(0,4)$, $\{ \alpha_{n}\},\{\beta_{n}\},\{\gamma_{n}\}\subset(0,1)$ satisfy the following conditions:

(i) $\lim_{n\rightarrow\infty}\beta_{n} =0$ and $\sum_{n=0}^{\infty}\beta_{n} = \infty$,
(ii) $\liminf_{n\rightarrow\infty} \alpha_{n} \gamma_{n} > 0$.

We observe that in the proof of their proposed results, the sequence $\{ \xi_{n}\}$ may not converges to the zero, for instance if $A=I$ and $\rho_{n} = 2$ for all $n=0,1,2,\ldots $ then the sequence $\{\xi_{n}\}$ converges to the integer 1. The relaxed *CQ*-like algorithms are also proposed subsequently [19–21].

In this paper, we modify in all these algorithms to solve the SCFP (1.6) and also solve the SFP (1.1) based on Moudafi’s viscosity approximation method [22], in the case that the firmly nonexpansive mappings *S* and *T* such that both $I-S$ and $I-T$ are demiclosed at zero, and an *α*-contraction mapping $h:H \rightarrow H$ with $\alpha\in(0,1)$ on an infinite-dimensional real Hilbert space in a single-step regularized method with the regularization parameter as follows:

1.7 $$ x_{n+1} = \alpha_{n} x_{n} + \beta_{n} h(x_{n}) +\gamma_{n} S \bigl( I-\lambda _{n} \bigl(A^{*} (I-T)A+\epsilon_{n} I \bigr) \bigr)x_{n},\quad \forall n=0,1,2,\ldots. $$

We suggest and analyze this iterative scheme (1.7) under some appropriate conditions imposed on the parameters with a new step size, which is not depend on the related matrix inverses and the largest eigenvalue (or the spectral radius of the self-adjoint operator) of the related matrix such that another strong convergence theorems for the SCFP (1.6) and the SFP (1.1) are obtained.

## Preliminaries

Let *H* and *K* be two real Hilbert spaces, $A:H\rightarrow K$ be a bounded linear operator, $A^{*}$ denotes the adjoint of *A* and let *I* be the identity operator on *H* or *K*. If $f: H \rightarrow\mathbb {R}$ is a differentiable function, then we denote ∇*f* the gradient of the function *f*. We will also use the notation: → to denote the strong convergency, ⇀ to denote the weak convergency,

$$\omega_{w}(x_{n}) = \bigl\{ x: \exists\{x_{n_{k}} \} \subset\{x_{n}\} \text{ such that } x_{n_{k}} \rightharpoonup x \bigr\} $$

to denote the weak limit set of $\{x_{n}\}$ and $\operatorname{Fix}(T) = \{ x:x=Tx \}$ to denote the fixed point set of the mapping *T*.

Let *C* be a nonempty closed convex subset of a real Hilbert space *H*. Recall that the metric projection $P_{C}: H \rightarrow C$ is defined as follows: for each $x \in H$, $P_{C} x$ is the unique point in *C* satisfying

$$\Vert x-P_{C} x \Vert = \inf\bigl\{ \Vert x-y \Vert :y\in C \bigr\} . $$

Let $f:H \rightarrow\mathbb{R}$ be a function. Recall that a function *f* is called convex if

$$f \bigl(\lambda x+(1-\lambda)y \bigr) \leq\lambda f(x) + (1-\lambda)f(y),\quad \forall \lambda\in[0,1], \forall x,y \in H. $$

A differentiable function *f* is convex if and only if for each $x\in H$, we have the inequality:

$$f(z) \geq f(x)+ \bigl\langle \nabla f(x),z-x \bigr\rangle , \quad\forall z \in H. $$

An element $g \in H$ is said to be a subgradient of *f* at $x\in H$ if we have the subdifferentiable inequality

$$f(z) \geq f(x)+ \langle g,z-x \rangle, \quad\forall z \in H. $$

A function *f* is said to be subdifferentiable at $x\in H$, if it has at least one subgradient at *x*. The set of subgradients of *f* at $x\in H$ is called the subdifferentiable of *f* at *x*, and it is denoted by $\partial f(x)$. A function *f* is called subdifferentiable, if it is subdifferentiable at all ${x\in H}$. If a function *f* is differentiable and convex, then its gradient and subgradient coincide. A function *f* is called lower semi-continuous (lsc) for all $x\in H$ if for each $a\in\mathbb{R}$, the set $\{x\in H: f(x) \leq a\}$ is a closed set, and a function *f* is called weakly lower semi-continuous (w-lsc) at $x\in H$ if *f* is lsc at *x* for a sequence $\{x_{n}\}\subset H$ such that $x_{n} \rightharpoonup x$.

We collect some known lemmas and definitions which are our main tools in proving the our results.

### Lemma 2.1

*Let* *H* *be a real Hilbert space*. *Then*, *for all* $x,y\in H$,

(i) $\|x+y\|^{2} = \|x\|^{2}+2 \langle x,y \rangle+\| y\|^{2}$,
(ii) $\|x+y\|^{2} \leq\|x\|^{2} + 2 \langle y,x+y \rangle$.

### Lemma 2.2

([23])

*Let* *C* *be a nonempty closed convex subset of a real Hilbert space* *H*. *Then*:

(i) $z=P_{C}x \Leftrightarrow \langle x-z,z -y \rangle\geq0$, $\forall x\in H$, $y \in C$,
(ii) $z=P_{C}x \Leftrightarrow\|x-z \|^{2} \leq\|x-y\|^{2} - \| y-z \|^{2}$, $\forall x\in H$, $y \in C$,
(iii) $\| P_{C} x - P_{C} y\|^{2} \leq \langle x-y,P_{C} x - P_{C} y \rangle$, $\forall x,y\in H$.

### Definition 2.3

Let *H* be a real Hilbert space. The operator $T:H\rightarrow H$ is called:

(i) monotone if
  $$\langle x-y,Tx-Ty \rangle\geq0,\quad \forall x,y \in H, $$
(ii) *L*-Lipschitzian with $L>0$ if
  $$\Vert Tx-Ty \Vert \leq L \Vert x-y \Vert ,\quad \forall x,y \in H, $$
(iii) *α*-contraction if it is *α*-Lipschitzian with $\alpha\in(0,1)$,
(iv) nonexpansive if it is 1-Lipschitzian,
(v) firmly nonexpansive if
  $$\Vert Tx-Ty \Vert ^{2} \leq \Vert x-y \Vert ^{2} - \bigl\Vert (I-T)x-(I-T)y \bigr\Vert ^{2}, \quad\forall x,y\in H. $$

### Lemma 2.4

([24])

*Let* *H* *and* *K* *be two real Hilbert spaces and let* $T:K \rightarrow K$ *be a firmly nonexpansive mapping such that* $\|(I-T)x\|$ *is a convex function from* *K* *to* $\overline{\mathbb{R}}=[-\infty,+\infty]$. *Let* $A:H\rightarrow K$ *be a bounded linear operator and* $f(x) = \frac {1}{2}\|(I-T)Ax\|^{2} $ *for all* $x\in H$. *Then*:

(i) $\nabla f(x) = A^{*}(I-T)Ax$, $\forall x\in H$,
(ii) ∇*f* *is* $\|A\|^{2}$-*Lipschitzian*.

### Lemma 2.5

([24])

*Let* *H* *be a real Hilbert space and* $T: H\rightarrow H$ *be an operator*. *The following statements are equivalent*:

(i) *T* *is firmly nonexpansive*,
(ii) $\|Tx-Ty\|^{2} \leq \langle x-y,Tx-Ty \rangle$, $\forall x,y\in H$,
(iii) $I-T$ *is firmly nonexpansive*.

### Lemma 2.6

([25])

*Let* *H* *be a real Hilbert space and let* $\{x_{n}\}$ *be a sequence in* *H*. *Then*, *for any given sequence* $\{\lambda_{n} \}_{n=1}^{\infty}\subset (0,1)$ *with* $\sum_{n=1}^{\infty}\lambda_{n} = 1$ *and for any positive integer* *i*, *j* *with* $i< j$,

$$\Biggl\Vert \sum_{i=1}^{\infty}\lambda_{n} x_{n} \Biggr\Vert ^{2} \leq\sum _{i=1}^{\infty}\lambda_{n} \Vert x_{n} \Vert ^{2} - \lambda_{i} \lambda_{j} \Vert x_{i}-x_{j} \Vert ^{2}. $$

### Lemma 2.7

([26])

*Let* $\{a_{n}\}$ *be a sequence of nonnegative real numbers such that*

$$a_{n+1} \leq(1-\gamma_{n})a_{n} + \gamma_{n} \sigma_{n},\quad \forall n=0,1,2,\ldots, $$

*where* $\{\gamma_{n}\}$ *is a sequence in* $(0,1)$ *and* $\{\sigma_{n} \}$ *is a sequence in* $\mathbb{R}$ *such that*

(i) $\sum_{n=0}^{\infty}\gamma_{n} = \infty$,
(ii) $\limsup_{n\rightarrow\infty} \sigma_{n} \leq0$ *or* $\sum_{n=0}^{\infty}|\gamma_{n} \sigma_{n}| < \infty$.

*Then* $\lim_{n\rightarrow\infty}a_{n} = 0$.

### Lemma 2.8

([27])

*Let* $\{t_{n}\}$ *be a sequence of real numbers such that there exists a subsequence* $\{n_{i}\}$ *of* $\{n\}$ *such that* $t_{n_{i}} < t_{n_{i}+1}$ *for all* $i \in\mathbb{N}$. *Then there exists a nondecreasing sequence* $\{ \tau(n) \} \subset\mathbb{N}$ *such that* $\tau(n) \rightarrow\infty $, *and the following properties are satisfied by all* (*sufficiently large*) *numbers* $n \in\mathbb{N}$:

$$t_{\tau(n)} \leq t_{\tau(n)+1},\qquad t_{n} \leq t_{\tau(n)+1}. $$

*In fact*,

$$\tau(n) = \max\{k\leq n:t_{k} < t_{k+1} \}. $$

### Lemma 2.9

([28] (Demiclosedness principle))

*Let* *C* *be a nonempty closed convex subset of a real Hilbert space* *H* *and let* $S:C \rightarrow C$ *be a nonexpansive mapping with* $\operatorname {Fix}(S)\neq\emptyset$. *If the sequence* $\{x_{n}\}\subset C$ *converges weakly to* *x* *and the sequence* $\{(I-S)x_{n}\}$ *converges strongly to* *y*. *Then* $(I-S)x = y$; *in particular*, *if* $y=0$ *then* $x\in\operatorname{Fix}(S)$.

### Lemma 2.10

([16])

*Let* *C* *be a nonempty closed convex subset of a Hilbert space* *H* *and let* *f* *be a proper convex lower semi*-*continuous function of* *C* *into* $(-\infty,\infty]$. *If* $\{x_{n}\}$ *be a bounded sequence of* *C* *such that* $x_{n}\rightharpoonup x_{0}$. *Then* $f(x_{0}) \leq\liminf_{n\rightarrow \infty} f(x_{n})$.

## Main result

Throughout this paper, we let $\Omega:= \{x\in\operatorname{Fix}(S): Ax \in \operatorname{Fix}(T)\}$. It is clear that Ω is closed and convex.

### Theorem 3.1

*Let* *H* *and* *K* *be two real Hilbert spaces and let* $S:H \rightarrow H$ *and* $T:K \rightarrow K$ *be two firmly nonexpansive mappings such that both* $I-S$ *and* $I-T$ *are demiclosed at zero*. *Let* $\|(I-T)x\|$ *be a convex function from* *K* *to* $\overline{\mathbb{R}}$ *and* $A: H \rightarrow K$ *be a bounded linear operator*, *and let* $h:H\rightarrow H$ *be an* *α*-*contraction mapping*. *Assume that the SCFP* (1.6) *has a nonempty solution set* Ω *and let* $\{x_{n}\} \subset H$ *be a sequence generated by*

$$ \textstyle\begin{cases} x_{0} \in H, \\ x_{n+1} = \alpha_{n} x_{n}+\beta_{n} h(x_{n}) +\gamma_{n} S(x_{n}- \lambda_{n} \nabla g_{\epsilon_{n}}(x_{n}) ), \quad\forall n=0,1,2,\ldots, \end{cases} $$

*where* $\alpha_{n}+\beta_{n}+\gamma_{n} = 1$ *and* $g_{\epsilon_{n}}(x_{n}) = \frac{1}{2}\|(I-T)Ax_{n}\|^{2}+\frac{\epsilon_{n}}{2}\|x_{n}\|^{2}$ *such that*

$$\nabla g_{\epsilon_{n}}(x_{n}) = A^{*} (I-T)Ax_{n}+ \epsilon_{n} x_{n} \neq0 ,\qquad \lambda_{n} = \frac{\rho_{n} g_{\epsilon_{n}}(x_{n})}{ \Vert \nabla g_{\epsilon _{n}}(x_{n}) \Vert ^{2} + \Vert \nabla g_{\epsilon_{n}}(x_{n}) \Vert +\rho_{n} g_{\epsilon_{n}}(x_{n})} $$

*for all* $n=0,1,2,\ldots$ . *If the sequences* $\{\rho_{n} \}\subset[a,b]$ *for some* $a,b \in(0,2)$, $\{ \alpha_{n} \} \subset[c,d]$ *for some* $c,d \in (0,1)$ *and* $\{\beta_{n} \},\{\gamma_{n} \}\subset(0,1)$ *satisfy the following conditions*:

(i) $0 \leq\epsilon_{n} \leq\beta_{n}^{2m}$ *such that* $m>1$ *for all* $n=0,1,2,\ldots $ ,
(ii) $\lim_{n\rightarrow\infty}\beta_{n} = 0$ *and* $\sum_{n=0}^{\infty}\beta_{n} = \infty$,

*then the sequence* $\{x_{n}\}$ *converges strongly to* $x^{*} \in\Omega$ *where* $x^{*} = P_{\Omega}h(x^{*})$.

### Proof

Fix $n\in\mathbb{N}\cup\{0\}$. Note that $g_{\epsilon_{n}}(x)= \frac {1}{2}\|(I-T)Ax\|^{2}+\frac{\epsilon_{n}}{2}\|x\|^{2}$ is Gáteaux differentiable by the convexity of $\|(I-T)Ax\|$ for all $x\in H$. First, we show that $\{x_{n}\}$ is bounded. Pick $p \in\Omega $. We have $p \in\operatorname{Fix}(S)$ and $Ap \in\operatorname{Fix}(T)$. Observing that $I-T$ is firmly nonexpansive, by Lemma 2.5 we have

3.1 $$\begin{aligned} \bigl\langle x_{n}-p,\nabla g_{\epsilon_{n}}(x_{n}) \bigr\rangle &= \bigl\langle x_{n}-p,A^{*}(I-T)Ax_{n}+ \epsilon_{n} x_{n} \bigr\rangle \\ &= \bigl\langle x_{n}-p,A^{*}(I-T)Ax_{n} \bigr\rangle + \epsilon_{n} \langle x_{n}-p,x_{n} \rangle \\ &= \bigl\langle Ax_{n}-Ap,(I-T)Ax_{n}-(I-T)Ap \bigr\rangle \\ &\quad{} +\frac{\epsilon_{n}}{2} \bigl( \Vert x_{n}-p \Vert ^{2}+ \Vert x_{n} \Vert ^{2}- \Vert p \Vert ^{2} \bigr) \\ &\geq \bigl\Vert (I-T)Ax_{n}-(I-T)Ap \bigr\Vert ^{2}+ \frac{\epsilon_{n}}{2} \bigl( \Vert x_{n} \Vert ^{2}- \Vert p \Vert ^{2} \bigr) \\ &\geq \frac{1}{2} \bigl\Vert (I-T)Ax_{n} \bigr\Vert ^{2}+ \frac{\epsilon_{n}}{2} \bigl( \Vert x_{n} \Vert ^{2}- \Vert p \Vert ^{2} \bigr) \\ &\geq g_{\epsilon_{n}}(x_{n})-\frac{\beta_{n}^{2m}}{2} \Vert p \Vert ^{2}. \end{aligned}$$

Therefore, by the nonexpansiveness of *S* we have

3.2 $$ \begin{aligned}[b] &\bigl\Vert S \bigl(x_{n}-\lambda_{n} \nabla g_{\epsilon_{n}}(x_{n}) \bigr)-p \bigr\Vert ^{2} \\ &\quad= \bigl\Vert S \bigl(x_{n}-\lambda_{n} \nabla g_{\epsilon_{n}}(x_{n}) \bigr)-Sp \bigr\Vert ^{2} \\ &\quad\leq \bigl\Vert x_{n}-\lambda_{n} \nabla g_{\epsilon_{n}}(x_{n})-p \bigr\Vert ^{2} \\ &\quad= \Vert x_{n}-p \Vert ^{2} +\lambda_{n}^{2} \bigl\Vert \nabla g_{\epsilon_{n}}(x_{n}) \bigr\Vert ^{2}-2 \lambda_{n} \bigl\langle x_{n}-p,\nabla g_{\epsilon_{n}}(x_{n}) \bigr\rangle \\ &\quad\leq \Vert x_{n}-p \Vert ^{2} +\lambda_{n}^{2} \bigl\Vert \nabla g_{\epsilon_{n}}(x_{n}) \bigr\Vert ^{2} -2 \lambda_{n} g_{\epsilon_{n}}(x_{n})+ \lambda_{n} \beta_{n}^{2m} \Vert p \Vert ^{2} \\ &\quad\leq \Vert x_{n}-p \Vert ^{2} +\frac{\rho_{n}^{2} g_{\epsilon_{n}}^{2}(x_{n}) ( \Vert \nabla g_{\epsilon_{n}}(x_{n}) \Vert ^{2} + \Vert \nabla g_{\epsilon_{n}}(x_{n}) \Vert +\rho _{n} g_{\epsilon_{n}}(x_{n}) )}{ ( \Vert \nabla g_{\epsilon_{n}}(x_{n}) \Vert ^{2} + \Vert \nabla g_{\epsilon_{n}}(x_{n}) \Vert +\rho_{n} g_{\epsilon_{n}}(x_{n}) )^{2}} \\ & \qquad{} -\frac{2\rho_{n} g_{\epsilon_{n}}^{2}(x_{n})}{ \Vert \nabla g_{\epsilon _{n}}(x_{n}) \Vert ^{2} + \Vert \nabla g_{\epsilon_{n}}(x_{n}) \Vert +\rho_{n} g_{\epsilon _{n}}(x_{n})}+ \beta_{n}^{2m} \Vert p \Vert ^{2} \\ &\quad= \Vert x_{n}-p \Vert ^{2}-\rho_{n}(2- \rho_{n})\frac{g_{\epsilon_{n}}^{2}(x_{n})}{ \Vert \nabla g_{\epsilon_{n}}(x_{n}) \Vert ^{2} + \Vert \nabla g_{\epsilon_{n}}(x_{n}) \Vert +\rho _{n} g_{\epsilon_{n}}(x_{n})} +\beta_{n}^{2m} \Vert p \Vert ^{2} \\ &\quad\leq \Vert x_{n}-p \Vert ^{2}+\beta_{n}^{2m} \Vert p \Vert ^{2} \\ &\quad\leq \bigl( \Vert x_{n}-p \Vert +\beta_{n}^{m} \Vert p \Vert \bigr)^{2} .\end{aligned} $$

This implies that

3.3 $$ \bigl\Vert S \bigl(x_{n}-\lambda_{n} \nabla g_{\epsilon_{n}}(x_{n}) \bigr)-p \bigr\Vert \leq \Vert x_{n}-p \Vert + \beta_{n}^{m} \Vert p \Vert . $$

Hence,

$$\begin{aligned} \Vert x_{n+1}-p \Vert &= \bigl\Vert \alpha_{n} x_{n}+ \beta_{n} h(x_{n})+\gamma_{n} S \bigl(x_{n}-\lambda_{n} \nabla g_{\epsilon_{n}}(x_{n}) \bigr)-p \bigr\Vert \\ &\leq \alpha_{n} \Vert x_{n}-p \Vert + \beta_{n} \bigl\Vert h(x_{n})-p \bigr\Vert + \gamma_{n} \bigl\Vert S \bigl(x_{n}- \lambda_{n} \nabla g_{\epsilon_{n}}(x_{n}) \bigr)-p \bigr\Vert \\ &\leq \alpha_{n} \Vert x_{n}-p \Vert + \beta_{n} \bigl\Vert h(x_{n})-h(p) \bigr\Vert + \beta_{n} \bigl\Vert h(p)-p \bigr\Vert \\ &\quad{} +\gamma_{n} \bigl( \Vert x_{n}-p \Vert + \beta_{n}^{m} \Vert p \Vert \bigr) \\ &\leq \alpha_{n} \Vert x_{n}-p \Vert + \beta_{n} \alpha \Vert x_{n}-p \Vert + \beta_{n} \bigl\Vert h(p)-p \bigr\Vert +\gamma_{n} \Vert x_{n}-p \Vert +\beta_{n} \Vert p \Vert \\ &= \bigl(1-(1-\alpha)\beta_{n} \bigr) \Vert x_{n}-p \Vert +(1- \alpha)\beta_{n} \frac{ \Vert h(p)-p \Vert + \Vert p \Vert }{1-\alpha} \\ &\leq \max \biggl\{ \Vert x_{n}-p \Vert ,\frac{ \Vert h(p)-p \Vert + \Vert p \Vert }{1-\alpha} \biggr\} .\end{aligned} $$

By induction, we have

$$\Vert x_{n}-p \Vert \leq\max \biggl\{ \Vert x_{0}-p \Vert ,\frac{ \Vert h(p)-p \Vert + \Vert p \Vert }{1-\alpha} \biggr\} . $$

This implies that $\{x_{n}\}$ is bounded, and so are $\{h(x_{n})\}$ and $\{ g_{\epsilon_{n}}(x_{n})\}$. Using Lemma 2.6 and (3.2), we have

$$\begin{aligned} \Vert x_{n+1}-p \Vert ^{2} &= \bigl\Vert \alpha_{n} x_{n}+\beta_{n} h(x_{n})+ \gamma_{n} S \bigl(x_{n}-\lambda_{n} \nabla g_{\epsilon_{n}}(x_{n}) \bigr)-p \bigr\Vert ^{2} \\ &= \bigl\Vert \alpha_{n} (x_{n}-p)+\beta_{n} \bigl(h(x_{n})-p \bigr)+\gamma_{n} \bigl(S \bigl(x_{n}-\lambda _{n} \nabla g_{\epsilon_{n}}(x_{n}) \bigr)-p \bigr) \bigr\Vert ^{2} \\ &\leq \alpha_{n} \Vert x_{n}-p \Vert ^{2} + \beta_{n} \bigl\Vert h(x_{n})-p \bigr\Vert ^{2} + \gamma_{n} \bigl\Vert S \bigl(x_{n}- \lambda_{n} \nabla g_{\epsilon_{n}}(x_{n}) \bigr)-p \bigr\Vert ^{2} \\ &\quad{} -\alpha_{n} \gamma_{n} \bigl\Vert S \bigl(x_{n}- \lambda_{n} \nabla g_{\epsilon _{n}}(x_{n}) \bigr)-x_{n} \bigr\Vert ^{2} \\ &\leq \alpha_{n} \Vert x_{n}-p \Vert ^{2} + \beta_{n} \bigl\Vert h(x_{n})-p \bigr\Vert ^{2} + \gamma_{n} \biggl( \Vert x_{n}-p \Vert ^{2} \\ &\quad{} -\rho_{n}(2-\rho_{n})\frac{g_{\epsilon_{n}}^{2}(x_{n})}{ \Vert \nabla g_{\epsilon_{n}}(x_{n}) \Vert ^{2} + \Vert \nabla g_{\epsilon_{n}}(x_{n}) \Vert +\rho_{n} g_{\epsilon_{n}}(x_{n})} + \beta_{n}^{2m} \Vert p \Vert ^{2} \biggr) \\ &\quad{} -\alpha_{n} \gamma_{n} \bigl\Vert S \bigl(x_{n}- \lambda_{n} \nabla g_{\epsilon _{n}}(x_{n}) \bigr)-x_{n} \bigr\Vert ^{2} \\ &\leq \Vert x_{n}-p \Vert ^{2} +\beta_{n} \bigl\Vert h(x_{n})-p \bigr\Vert ^{2} \\ &\quad{} -\gamma_{n} \rho_{n}(2-\rho_{n}) \frac{g_{\epsilon_{n}}^{2}(x_{n})}{ \Vert \nabla g_{\epsilon_{n}}(x_{n}) \Vert ^{2} + \Vert \nabla g_{\epsilon_{n}}(x_{n}) \Vert +\rho_{n} g_{\epsilon_{n}}(x_{n})}+\beta_{n} \Vert p \Vert ^{2} \\ &\quad{} -\alpha_{n} \gamma_{n} \bigl\Vert S \bigl(x_{n}-\lambda_{n} \nabla g_{\epsilon_{n}}(x_{n}) \bigr)-x_{n} \bigr\Vert ^{2} .\end{aligned} $$

Therefore,

3.4 $$ \begin{aligned}[b]& \gamma_{n} \rho_{n}(2- \rho_{n})\frac{g_{\epsilon_{n}}^{2}(x_{n})}{ \Vert \nabla g_{\epsilon_{n}}(x_{n}) \Vert ^{2} + \Vert \nabla g_{\epsilon_{n}}(x_{n}) \Vert +\rho_{n} g_{\epsilon_{n}}(x_{n})} \\ &\qquad{} +\alpha_{n} \gamma_{n} \bigl\Vert S \bigl(x_{n}- \lambda_{n} \nabla g_{\epsilon _{n}}(x_{n}) \bigr)-x_{n} \bigr\Vert ^{2} \\ &\quad\leq \Vert x_{n}-p \Vert ^{2} - \Vert x_{n+1}-p \Vert ^{2} +\beta_{n} \bigl\Vert h(x_{n})-p \bigr\Vert ^{2}+ \beta_{n} \Vert p \Vert ^{2}.\end{aligned} $$

Since $P_{\Omega}h$ is a contraction on *H*, by the Banach contraction principle there exists a unique element $x^{*} \in H$ such that $x^{*} = P_{\Omega}h(x^{*})$. That is $x^{*} \in\Omega$. Now, we show that $x_{n} \rightarrow x^{*}$ as $n\rightarrow\infty$. We consider into two cases.

Case 1. Assume that $\{\|x_{n}-p\|\}$ is a monotone sequence. In other words, for $n_{0}$ large enough, $\{\|x_{n}-p\|\}_{n\geq n_{0}}$ is either nondecreasing or nonincreasing. As $\{\|x_{n}-p\|\}$ is bounded, so $\{\| x_{n}-p\|\}$ is convergent. Therefore, by (3.4) we have

3.5 $$ \lim_{n\rightarrow\infty} \gamma_{n} \rho_{n}(2-\rho_{n})\frac {g_{\epsilon_{n}}^{2}(x_{n})}{ \Vert \nabla g_{\epsilon_{n}}(x_{n}) \Vert ^{2} + \Vert \nabla g_{\epsilon_{n}}(x_{n}) \Vert +\rho_{n} g_{\epsilon_{n}}(x_{n})} = 0 $$

and

3.6 $$ \lim_{n\rightarrow\infty} \alpha_{n} \gamma_{n} \bigl\Vert S \bigl(x_{n}-\lambda_{n} \nabla g_{\epsilon_{n}}(x_{n}) \bigr)-x_{n} \bigr\Vert ^{2} =0. $$

By Lemma 2.4, we have

$$\begin{aligned} \bigl\Vert \nabla g_{\epsilon_{n}}(x_{n}) \bigr\Vert &\leq \bigl\Vert \nabla g_{\epsilon_{n}}(x_{n})-\nabla g_{\epsilon_{n}}(p) \bigr\Vert + \bigl\Vert \nabla g_{\epsilon_{n}}(p) \bigr\Vert \\ &\leq \bigl\Vert A^{*}(I-T)Ax_{n}-A^{*}(I-T)Ap \bigr\Vert + \epsilon_{n} \Vert x_{n}-p \Vert + \bigl\Vert \nabla g_{\epsilon_{n}}(p) \bigr\Vert \\ &\leq \bigl( \Vert A \Vert ^{2}+\epsilon_{n} \bigr) \Vert x_{n}-p \Vert + \bigl\Vert \nabla g_{\epsilon_{n}}(p) \bigr\Vert \\ &\leq \bigl( \Vert A \Vert ^{2}+1 \bigr) \Vert x_{n}-p \Vert + \bigl\Vert \nabla g_{\epsilon_{n}}(p) \bigr\Vert .\end{aligned} $$

This implies that $\{\|\nabla g_{\epsilon_{n}}(x_{n})\|\}$ is bounded, and so is $\{\|\nabla g_{\epsilon_{n}}(x_{n})\|^{2}+\|\nabla g_{\epsilon _{n}}(x_{n})\|+\rho_{n} g_{\epsilon_{n}}(x_{n})\}$. Hence, $\|\nabla g_{\epsilon_{n}}(x_{n})\|^{2}+\|\nabla g_{\epsilon_{n}}(x_{n})\|+\rho_{n} g_{\epsilon_{n}}(x_{n}) < \delta$ for some $\delta>0$. Since

$$\begin{gathered} (1-d-\beta_{n}) a (2-b)\frac{g_{\epsilon_{n}}^{2}(x_{n})}{\delta} \\ \quad\leq \gamma_{n} \rho_{n}(2-\rho_{n}) \frac{g_{\epsilon_{n}}^{2}(x_{n})}{ \Vert \nabla g_{\epsilon_{n}}(x_{n}) \Vert ^{2} + \Vert \nabla g_{\epsilon_{n}}(x_{n}) \Vert +\rho _{n} g_{\epsilon_{n}}(x_{n})},\end{gathered} $$

by (3.5) we have

3.7 $$ \lim_{n\rightarrow\infty} g_{\epsilon_{n}}(x_{n}) = 0. $$

Since

$$c (1-d-\beta_{n}) \bigl\Vert S \bigl(x_{n}- \lambda_{n} \nabla g_{\epsilon_{n}}(x_{n}) \bigr)-x_{n} \bigr\Vert ^{2} \leq\alpha_{n} \gamma_{n} \bigl\Vert S \bigl(x_{n}- \lambda_{n} \nabla g_{\epsilon _{n}}(x_{n}) \bigr)-x_{n} \bigr\Vert ^{2}, $$

by (3.6) we have

3.8 $$ \lim_{n\rightarrow\infty} \bigl\Vert S \bigl(x_{n}- \lambda_{n} \nabla g_{\epsilon _{n}}(x_{n}) \bigr)-x_{n} \bigr\Vert =0. $$

Here we have $\lim_{n\rightarrow\infty} \epsilon_{n} = 0$ by the condition (ii). Therefore, in the same way, we have

$$\lim_{n\rightarrow\infty} \bigl\Vert (I-T)Ax_{n} \bigr\Vert = 0. $$

Consider a subsequence $\{x_{n_{k}}\}$ of $\{x_{n}\}$. As $\{x_{n}\}$ is bounded, so $\{x_{n_{k}}\}$ is bounded, there exists a subsequence $\{ x_{n_{k_{l}}}\}$ of $\{x_{n_{k}}\}$ which converges weakly to $w\in H$. Without loss of generality, we can assume that $x_{n_{k}} \rightharpoonup w$ as $k\rightarrow\infty$. Therefore, $Ax_{n_{k}} \rightharpoonup Aw$ as $k\rightarrow\infty$ and

$$\lim_{k\rightarrow\infty} \bigl\Vert (I-T)Ax_{n_{k}} \bigr\Vert = 0. $$

By the demiclosedness at zero, we have $Aw \in\operatorname{Fix}(T)$. Next, we show that $w \in\operatorname{Fix}(S)$. By Lemma 2.1(2), the firmly nonexpansiveness of *S* and (3.3), we have

$$\begin{aligned}& \bigl\Vert S \bigl(x_{n}-\lambda_{n} \nabla g_{\epsilon_{n}}(x_{n}) \bigr)-p \bigr\Vert ^{2} \\& \quad= \bigl\Vert \bigl(S \bigl(x_{n}-\lambda_{n} \nabla g_{\epsilon_{n}}(x_{n}) \bigr)-Sx_{n} \bigr) +(Sx_{n} -Sp) \bigr\Vert ^{2} \\& \quad\leq \Vert Sx_{n}-Sp \Vert ^{2}+2 \bigl\langle S \bigl(x_{n}-\lambda_{n} \nabla g_{\epsilon_{n}}(x_{n}) \bigr)-Sx_{n},S \bigl(x_{n}-\lambda_{n} \nabla g_{\epsilon _{n}}(x_{n}) \bigr)-p \bigr\rangle \\& \quad\leq \Vert x_{n}-p \Vert ^{2}- \bigl\Vert (I-S)x_{n}-(I-S)p \bigr\Vert ^{2} \\& \qquad{}+2 \bigl\Vert S \bigl(x_{n}-\lambda_{n} \nabla g_{\epsilon_{n}}(x_{n}) \bigr)-Sx_{n} \bigr\Vert \bigl\Vert S \bigl(x_{n}-\lambda_{n} \nabla g_{\epsilon_{n}}(x_{n}) \bigr)-p \bigr\Vert \\& \quad\leq \Vert x_{n}-p \Vert ^{2}- \bigl\Vert (I-S)x_{n} \bigr\Vert ^{2} +2\lambda_{n} \bigl\Vert \nabla g_{\epsilon _{n}}(x_{n}) \bigr\Vert \bigl( \Vert x_{n}-p \Vert +\beta_{n}^{m} \Vert p \Vert \bigr). \end{aligned}$$

Therefore,

$$\begin{gathered} \bigl\Vert (I-S)x_{n} \bigr\Vert ^{2} \\ \quad\leq \Vert x_{n}-p \Vert ^{2} - \bigl\Vert S \bigl(x_{n}- \lambda_{n} \nabla g_{\epsilon _{n}}(x_{n}) \bigr)-p \bigr\Vert ^{2} +2\lambda_{n} \bigl\Vert \nabla g_{\epsilon_{n}}(x_{n}) \bigr\Vert \bigl( \Vert x_{n}-p \Vert +\beta_{n}^{m} \Vert p \Vert \bigr) \\ \quad\leq \Vert x_{n}-p \Vert ^{2} - \bigl( \bigl\Vert S \bigl(x_{n}- \lambda_{n} \nabla g_{\epsilon _{n}}(x_{n}) \bigr)-x_{n} \bigr\Vert - \Vert x_{n}-p \Vert \bigr)^{2} \\ \qquad{}+2\lambda_{n} \bigl\Vert \nabla g_{\epsilon_{n}}(x_{n}) \bigr\Vert \bigl( \Vert x_{n}-p \Vert +\beta _{n} \Vert p \Vert \bigr) \\ \quad\leq 2 \bigl\Vert S \bigl(x_{n}-\lambda_{n} \nabla g_{\epsilon_{n}}(x_{n}) \bigr)-x_{n} \bigr\Vert \Vert x_{n}-p \Vert +2\lambda_{n} \bigl\Vert \nabla g_{\epsilon_{n}}(x_{n}) \bigr\Vert \bigl( \Vert x_{n}-p \Vert +\beta_{n} \Vert p \Vert \bigr) \\ \quad= 2 \bigl\Vert S \bigl(x_{n}-\lambda_{n} \nabla g_{\epsilon_{n}}(x_{n}) \bigr)-x_{n} \bigr\Vert \Vert x_{n}-p \Vert \\ \qquad{}+\frac{2\rho_{n} g_{\epsilon_{n}}(x_{n}) \Vert \nabla g_{\epsilon_{n}}(x_{n}) \Vert }{ \Vert \nabla g_{\epsilon_{n}}(x_{n}) \Vert ^{2}+ \Vert \nabla g_{\epsilon_{n}}(x_{n}) \Vert +\rho_{n} g_{\epsilon_{n}}(x_{n}) } \bigl( \Vert x_{n}-p \Vert + \beta_{n} \Vert p \Vert \bigr) \\ \quad\leq 2 \bigl\Vert S \bigl(x_{n}-\lambda_{n} \nabla g_{\epsilon_{n}}(x_{n}) \bigr)-x_{n} \bigr\Vert \Vert x_{n}-p \Vert +4g_{\epsilon_{n}}(x_{n}) \bigl( \Vert x_{n}-p \Vert + \beta_{n} \Vert p \Vert \bigr).\end{gathered} $$

It follows by (3.7) and (3.8) that

$$\lim_{n\rightarrow\infty} \bigl\Vert (I-S)x_{n} \bigr\Vert = 0. $$

Hence, $\lim_{k\rightarrow\infty} \|(I-S)x_{n_{k}}\| = 0$. Therefore, by the demiclosedness at zero, we have $w\in\operatorname{Fix}(S)$. That is $w \in\Omega$. Applying the characteristic of $P_{\Omega}$ in Lemma 2.2(i) and $x^{*} = P_{\Omega}h(x^{*})$, we have

$$\limsup_{n\rightarrow\infty} \bigl\langle h \bigl(x^{*} \bigr)-x^{*},x_{n+1}-x^{*} \bigr\rangle = \max_{w\in\omega_{w} (x_{n})} \bigl\langle h \bigl(x^{*} \bigr)-x^{*} , w-x^{*} \bigr\rangle \leq0. $$

Finally, we show that $x_{n} \rightarrow x^{*}$ as $n \rightarrow\infty$. By Lemma 2.1(ii) and (3.3), we have

$$\begin{aligned} \bigl\Vert x_{n+1}-x^{*} \bigr\Vert ^{2} &= \bigl\Vert \alpha_{n} x_{n}+\beta_{n} h(x_{n})+ \gamma_{n} S \bigl(x_{n}-\lambda_{n} \nabla g_{\epsilon_{n}}(x_{n}) \bigr)-x^{*} \bigr\Vert ^{2} \\ &= \bigl\Vert \alpha_{n} \bigl(x_{n}-x^{*} \bigr)+ \beta_{n} \bigl( h(x_{n})-x^{*} \bigr)+\gamma_{n} \bigl( S \bigl(x_{n}-\lambda_{n} \nabla g_{\epsilon_{n}}(x_{n}) \bigr)-x^{*} \bigr) \bigr\Vert ^{2} \\ &\leq \bigl\Vert \alpha_{n} \bigl(x_{n}-x^{*} \bigr)+ \gamma_{n} \bigl( S \bigl(x_{n}-\lambda_{n} \nabla g_{\epsilon_{n}}(x_{n}) \bigr)-x^{*} \bigr) \bigr\Vert ^{2} \\ &\quad{} +2\beta_{n} \bigl\langle h(x_{n})-x^{*},x_{n+1}-x^{*} \bigr\rangle \\ &\leq \bigl( \alpha_{n} \bigl\Vert x_{n}-x^{*} \bigr\Vert + \gamma_{n} \bigl\Vert S \bigl(x_{n}- \lambda_{n} \nabla g_{\epsilon_{n}}(x_{n}) \bigr)-x^{*} \bigr\Vert \bigr)^{2} \\ &\quad{} +2\beta_{n} \bigl\langle h(x_{n})-h \bigl(x^{*} \bigr),x_{n+1}-x^{*} \bigr\rangle +2\beta_{n} \bigl\langle h \bigl(x^{*} \bigr)-x^{*},x_{n+1}-x^{*} \bigr\rangle \\ &\leq\bigl( \alpha_{n} \bigl\Vert x_{n}-x^{*} \bigr\Vert + \gamma_{n} \bigl( \bigl\Vert x_{n}-x^{*} \bigr\Vert +\beta _{n}^{m} \bigl\Vert x^{*} \bigr\Vert \bigr) \bigr)^{2} \\ &\quad{} +2\beta_{n} \alpha \bigl\Vert x_{n}-x^{*} \bigr\Vert \bigl\Vert x_{n+1}-x^{*} \bigr\Vert +2\beta_{n} \bigl\langle h \bigl(x^{*} \bigr)-x^{*},x_{n+1}-x^{*} \bigr\rangle \\ &\leq \bigl( (1-\beta_{n}) \bigl\Vert x_{n}-x^{*} \bigr\Vert + \beta_{n}^{m} \bigl\Vert x^{*} \bigr\Vert \bigr)^{2} + \beta_{n} \alpha \bigl( \bigl\Vert x_{n}-x^{*} \bigr\Vert ^{2} + \bigl\Vert x_{n+1}-x^{*} \bigr\Vert ^{2} \bigr) \\ &\quad{} +2\beta_{n} \bigl\langle h \bigl(x^{*} \bigr)-x^{*},x_{n+1}-x^{*} \bigr\rangle .\end{aligned} $$

This implies that

$$\begin{aligned} \bigl\Vert x_{n+1}-x^{*} \bigr\Vert ^{2} &\leq \frac{(1-\beta_{n})^{2}+\beta_{n} \alpha}{1-\beta_{n} \alpha} \bigl\Vert x_{n}-x^{*} \bigr\Vert ^{2}+ \frac{1}{1-\beta_{n} \alpha} \bigl( 2(1- \beta_{n})\beta_{n}^{m} \bigl\Vert x_{n}-x^{*} \bigr\Vert \bigl\Vert x^{*} \bigr\Vert \\ &\quad{} +\beta_{n}^{2m} \bigl\Vert x^{*} \bigr\Vert ^{2} +2 \beta_{n} \bigl\langle h \bigl(x^{*} \bigr)-x^{*},x_{n+1}-x^{*} \bigr\rangle \bigr) \\ &\leq\biggl( 1-\frac{2(1-\alpha)\beta_{n}}{1-\beta_{n} \alpha} \biggr) \bigl\Vert x_{n}-x^{*} \bigr\Vert ^{2}+ \frac{(1-\alpha)\beta_{n}}{1-\beta_{n} \alpha} \biggl( \frac{\beta_{n}}{1-\alpha} \bigl\Vert x_{n}-x^{*} \bigr\Vert ^{2} \\ &\quad{} +\frac{2\beta_{n}^{m-1}}{1-\alpha} \bigl\Vert x_{n}-x^{*} \bigr\Vert \bigl\Vert x^{*} \bigr\Vert +\frac{\beta _{n}^{2m-1}}{1-\alpha} \bigl\Vert x^{*} \bigr\Vert ^{2} +\frac{2}{1-\alpha} \bigl\langle h \bigl(x^{*} \bigr)-x^{*},x_{n+1}-x^{*} \bigr\rangle \biggr) \\ &\leq \biggl( 1-\frac{(1-\alpha)\beta_{n}}{1-\beta_{n} \alpha} \biggr) \bigl\Vert x_{n}-x^{*} \bigr\Vert ^{2}+ \frac{(1-\alpha)\beta_{n}}{1-\beta_{n} \alpha} \biggl( \frac{\beta_{n}}{1-\alpha}M^{2} \\ &\quad{} +\frac{2\beta_{n}^{m-1}}{1-\alpha}M \bigl\Vert x^{*} \bigr\Vert +\frac{\beta _{n}^{2m-1}}{1-\alpha} \bigl\Vert x^{*} \bigr\Vert ^{2} + \frac{2}{1-\alpha} \bigl\langle h \bigl(x^{*} \bigr)-x^{*},x_{n+1}-x^{*} \bigr\rangle \biggr) \\ &= (1-\eta_{n}) \bigl\Vert x_{n}-x^{*} \bigr\Vert ^{2}+ \eta_{n} \delta_{n},\end{aligned} $$

where $M= \sup_{n \geq0} \|x_{n}-x^{*}\|<\infty$, $\eta_{n} = \frac {(1-\alpha)\beta_{n}}{1-\beta_{n} \alpha}\in(0,1)$ and

$$\delta_{n} = \frac{\beta_{n}}{1-\alpha}M^{2} +\frac{2\beta _{n}^{m-1}}{1-\alpha}M \bigl\Vert x^{*} \bigr\Vert +\frac{\beta_{n}^{2m-1}}{1-\alpha} \bigl\Vert x^{*} \bigr\Vert ^{2} +\frac{2}{1-\alpha} \bigl\langle h \bigl(x^{*} \bigr)-x^{*},x_{n+1}-x^{*} \bigr\rangle . $$

It is easy to see that $\sum_{n=0}^{\infty}\eta_{n} = \infty$ and $\limsup_{n\rightarrow\infty} \delta_{n} \leq0$. Hence, by Lemma 2.7, the sequence $\{x_{n}\}$ converges strongly to $x^{*} = P_{\Omega}h(x^{*})$.

Case 2. Assume that $\{\|x_{n}-p\|\}$ is not a monotone sequence. Then we can define an integer sequence $\{\tau(n)\}$ for all $n \geq n_{0}$ (for some $n_{0}$ large enough) by

$$\tau(n) = \max\bigl\{ k\in\mathbb{N},k\leq n: \Vert x_{k}-p \Vert < \Vert x_{k+1}-p \Vert \bigr\} . $$

Clearly, $\{\tau(n)\}$ is a nondecreasing sequence such that $\tau(n) \rightarrow\infty$ as $n\rightarrow\infty$, and for all $n \geq n_{0}$ we have

$$\bigl\Vert x_{\tau(n)}-x^{*} \bigr\Vert < \bigl\Vert x_{\tau(n)+1}-x^{*} \bigr\Vert . $$

From (3.4), we obtain

$$\begin{gathered} \gamma_{\tau(n)} \rho_{\tau(n)}(2-\rho_{\tau(n)}) \frac {g_{\epsilon_{\tau(n)}}^{2}(x_{\tau(n)})}{ \Vert \nabla g_{\epsilon_{\tau (n)}}(x_{\tau(n)}) \Vert ^{2} + \Vert \nabla g_{\epsilon_{\tau(n)}}(x_{\tau (n)}) \Vert +\rho_{\tau(n)} g_{\epsilon_{\tau(n)}}(x_{\tau(n)})} \\ \qquad{} +\alpha_{\tau(n)} \gamma_{\tau(n)} \bigl\Vert S \bigl(x_{\tau(n)}- \lambda _{\tau(n)} \nabla g_{\epsilon_{\tau(n)}}(x_{\tau(n)}) \bigr)-x_{\tau (n)} \bigr\Vert ^{2} \\ \quad\leq \bigl\Vert x_{\tau(n)}-x^{*} \bigr\Vert ^{2} - \bigl\Vert x_{{\tau(n)}+1}-x^{*} \bigr\Vert ^{2} +\beta _{\tau(n)} \bigl\Vert h(x_{\tau(n)})-x^{*} \bigr\Vert ^{2}+ \beta_{\tau(n)} \bigl\Vert x^{*} \bigr\Vert ^{2} \\ \quad\leq \beta_{\tau(n)} \bigl\Vert h(x_{\tau(n)})-x^{*} \bigr\Vert ^{2}+ \beta_{\tau(n)} \bigl\Vert x^{*} \bigr\Vert ^{2}.\end{gathered} $$

As $\lim_{n\rightarrow\infty}\beta_{\tau(n)} = 0$ and $\{h(x_{\tau (n)})\}$ is bounded, we get

$$\lim_{n\rightarrow\infty} \gamma_{\tau(n)} \rho_{\tau(n)}(2-\rho _{\tau(n)})\frac{g_{\epsilon_{\tau(n)}}^{2}(x_{\tau(n)})}{ \Vert \nabla g_{\epsilon_{\tau(n)}}(x_{\tau(n)}) \Vert ^{2} + \Vert \nabla g_{\epsilon_{\tau (n)}}(x_{\tau(n)}) \Vert +\rho_{\tau(n)} g_{\epsilon_{\tau(n)}}(x_{\tau (n)})} = 0 $$

and

$$\lim_{n\rightarrow\infty}\alpha_{\tau(n)} \gamma_{\tau(n)} \bigl\Vert S \bigl(x_{\tau(n)}-\lambda_{\tau(n)} \nabla g_{\epsilon_{\tau (n)}}(x_{\tau(n)}) \bigr)-x_{\tau(n)} \bigr\Vert ^{2} = 0. $$

Following similar arguments to that in Case 1, we have $\omega _{w}(x_{\tau(n)})\subset\Omega$. Applying the characteristic of $P_{\Omega}$ in Lemma 2.2(i) and $x^{*} = P_{\Omega}h(x^{*})$, we have

$$\limsup_{n\rightarrow\infty} \bigl\langle h \bigl(x^{*} \bigr)-x^{*},x_{\tau (n)+1}-x^{*} \bigr\rangle = \max_{w\in\omega_{w} (x_{\tau(n)})} \bigl\langle h \bigl(x^{*} \bigr)-x^{*} , w-x^{*} \bigr\rangle \leq0, $$

and by similar arguments, we have

$$\bigl\Vert x_{\tau(n)+1}-x^{*} \bigr\Vert ^{2} \leq(1- \eta_{\tau(n)}) \bigl\Vert x_{\tau(n)}-x^{*} \bigr\Vert ^{2}+\eta_{\tau(n)} \delta_{\tau(n)}, $$

where $\eta_{\tau(n)} \in(0,1)$, $\sum_{\tau(n)}^{\infty}\eta_{\tau (n)} = \infty$ and $\limsup_{n\rightarrow\infty} \delta_{\tau(n)} \leq0$. Hence, by Lemma 2.7, we have $\lim_{n\rightarrow \infty} \|x_{\tau(n)}-x^{*}\| = 0$, and then $\lim_{n\rightarrow \infty} \|x_{\tau(n)+1}-x^{*}\| = 0$. Thus, by Lemma 2.8, we have

$$0 \leq \bigl\Vert x_{n}-x^{*} \bigr\Vert \leq\max \bigl\{ \bigl\Vert x_{\tau(n)}-x^{*} \bigr\Vert , \bigl\Vert x_{n}-x^{*} \bigr\Vert \bigr\} \leq \bigl\Vert x_{\tau(n)+1}-x^{*} \bigr\Vert . $$

Therefore, the sequence $\{x_{n}\}$ converges strongly to $x^{*} = P_{\Omega}h(x^{*})$. This completes the proof. □

### Corollary 3.2

*Let* *H* *and* *K* *be two real Hilbert spaces and let* $S:H \rightarrow H$ *and* $T:K \rightarrow K$ *be two firmly nonexpansive mappings such that both* $I-S$ *and* $I-T$ *are demiclosed at zero*. *Let* $\|(I-T)x\|$ *be a convex function from* *K* *to* $\overline{\mathbb{R}}$ *and* $A: H \rightarrow K$ *be a bounded linear operator*, *and let* $h:H\rightarrow H$ *be an* *α*-*contraction mapping*. *Assume that the SCFP* (1.6) *has a nonempty solution set* Ω *and let* $\{x_{n}\} \subset H$ *be a sequence generated by*

$$ \textstyle\begin{cases} x_{0} \in H, \\ x_{n+1} = \alpha_{n} x_{n}+\beta_{n} h(x_{n}) +\gamma_{n} S(x_{n}- \lambda_{n} \nabla g(x_{n}) ), \quad\forall n=0,1,2,\ldots, \end{cases} $$

*where* $\alpha_{n}+\beta_{n}+\gamma_{n} = 1$ *and* $g(x_{n}) = \frac{1}{2}\| (I-T)Ax_{n}\|^{2}$ *such that*

$$\nabla g(x_{n}) = A^{*} (I-T)Ax_{n} \neq0 ,\qquad \lambda_{n} = \frac{\rho_{n} g(x_{n})}{ \Vert \nabla g(x_{n}) \Vert ^{2} + \Vert \nabla g(x_{n}) \Vert +\rho_{n} g(x_{n})} $$

*for all* $n=0,1,2,\ldots$ . *If the sequences* $\{\rho_{n} \}\subset[a,b]$ *for some* $a,b \in(0,2)$, $\{ \alpha_{n} \} \subset[c,d]$ *for some* $c,d \in (0,1)$ *and* $\{\beta_{n} \},\{\gamma_{n} \}\subset(0,1)$ *satisfy the following conditions*: $\lim_{n\rightarrow\infty}\beta_{n} = 0$ *and* $\sum_{n=0}^{\infty}\beta_{n} = \infty$, *then the sequence* $\{x_{n}\}$ *converges strongly to* $x^{*} \in\Omega$ *where* $x^{*} = P_{\Omega}h(x^{*})$.

Let $\Gamma:= \{x\in C: Ax \in Q\}$. It is clear that Γ is closed and convex. Take $S=P_{C}$ and $T=P_{Q}$ into Theorem 3.1. We have the following consequences.

### Corollary 3.3

*Let* *C* *and* *Q* *be two nonempty closed convex subsets of real Hilbert spaces* *H* *and* *K*, *respectively*. *Let* $A: H \rightarrow K$ *be a bounded linear operator and* $h:H\rightarrow H$ *be an* *α*-*contraction mapping*. *Assume that the SFP* (1.1) *has a nonempty solution set* Γ *and let* $\{x_{n}\} \subset H$ *be a sequence generated by*

$$ \textstyle\begin{cases} x_{0} \in H, \\ x_{n+1} = \alpha_{n} x_{n}+\beta_{n} h(x_{n}) +\gamma_{n} P_{C}(x_{n}- \lambda_{n} \nabla g_{\epsilon_{n}}(x_{n}) ), \quad\forall n=0,1,2,\ldots, \end{cases} $$

*where* $\alpha_{n}+\beta_{n}+\gamma_{n} = 1$ *and* $g_{\epsilon_{n}}(x_{n}) = \frac{1}{2}\|(I-P_{Q})Ax_{n}\|^{2}+\frac{\epsilon_{n}}{2}\|x_{n}\|^{2}$ *such that*

$$\nabla g_{\epsilon_{n}}(x_{n}) = A^{*} (I-P_{Q})Ax_{n}+ \epsilon_{n} x_{n} \neq0 ,\qquad \lambda_{n} = \frac{\rho_{n} g_{\epsilon_{n}}(x_{n})}{ \Vert \nabla g_{\epsilon _{n}}(x_{n}) \Vert ^{2} + \Vert \nabla g_{\epsilon_{n}}(x_{n}) \Vert +\rho_{n} g_{\epsilon_{n}}(x_{n})} $$

*for all* $n=0,1,2,\ldots$ . *If the sequences* $\{\rho_{n} \}\subset[a,b]$ *for some* $a,b \in(0,2)$, $\{ \alpha_{n} \} \subset[c,d]$ *for some* $c,d \in (0,1)$ *and* $\{\beta_{n} \},\{\gamma_{n} \}\subset(0,1)$ *satisfy the following conditions*:

(i) $0 \leq\epsilon_{n} \leq\beta_{n}^{2m}$ *such that* $m>1$ *for all* $n=0,1,2,\ldots $ ,
(ii) $\lim_{n\rightarrow\infty}\beta_{n} = 0$ *and* $\sum_{n=0}^{\infty}\beta_{n} = \infty$,

*then the sequence* $\{x_{n}\}$ *converges strongly to* $x^{*} \in\Gamma$ *where* $x^{*} = P_{\Gamma}h(x^{*})$.

### Corollary 3.4

*Let* *C* *and* *Q* *be two nonempty closed convex subsets of real Hilbert spaces* *H* *and* *K*, *respectively*. *Let* $A: H \rightarrow K$ *be a bounded linear operator and* $h:H\rightarrow H$ *be an* *α*-*contraction mapping*. *Assume that the SFP* (1.1) *has a nonempty solution set* Γ *and let* $\{x_{n}\} \subset H$ *be a sequence generated by*

$$ \textstyle\begin{cases} x_{0} \in H, \\ x_{n+1} = \alpha_{n} x_{n}+\beta_{n} h(x_{n}) +\gamma_{n} P_{C}(x_{n}- \lambda_{n} \nabla g(x_{n}) ), \quad\forall n=0,1,2,\ldots, \end{cases} $$

*where* $\alpha_{n}+\beta_{n}+\gamma_{n} = 1$ *and* $g(x_{n}) = \frac{1}{2}\| (I-P_{Q})Ax_{n}\|^{2}$ *such that*

$$\nabla g(x_{n}) = A^{*} (I-P_{Q})Ax_{n}\neq0,\qquad \lambda_{n} = \frac{\rho_{n} g(x_{n})}{ \Vert \nabla g(x_{n}) \Vert ^{2} + \Vert \nabla g(x_{n}) \Vert +\rho_{n} g(x_{n})} $$

*for all* $n=0,1,2,\ldots$ . *If the sequences* $\{\rho_{n} \}\subset[a,b]$ *for some* $a,b \in(0,2)$, $\{ \alpha_{n} \} \subset[c,d]$ *for some* $c,d \in (0,1)$ *and* $\{\beta_{n} \},\{\gamma_{n} \}\subset(0,1)$ *satisfy the following conditions*: $\lim_{n\rightarrow\infty}\beta_{n} = 0$ *and* $\sum_{n=0}^{\infty}\beta_{n} = \infty$, *then the sequence* $\{x_{n}\}$ *converges strongly to* $x^{*} \in\Gamma$ *where* $x^{*} = P_{\Gamma}h(x^{*})$.

## Applications

In this section, assuming that the projections $P_{C}$ and $P_{Q}$ are not easily calculated. We present a perturbation technique. Carefully speaking, the convex sets *C* and *Q* satisfy the following assumptions:

1. The sets *C* and *Q* are given by
  $$\begin{gathered} C= \bigl\{ x\in H : c(x) \leq0 \bigr\} \neq\emptyset, \\ Q= \bigl\{ y\in K : q(y) \leq0 \bigr\} \neq\emptyset,\end{gathered} $$
  where $c:H\rightarrow\mathbb{R}$ and $q:K\rightarrow\mathbb{R}$ are two convex (not necessarily differentiable) functions.
2. For any $x\in H$, at least one subgradient $\xi\in \partial c(x)$ can be calculated, and for any $y\in K$, at least one subgradient $\eta\in\partial q(y)$ can be calculated, where $\partial c(x)$ and $\partial q(y)$ are a generalized gradient of $c(x)$ at *x* and a generalized gradient of $q(y)$ at *y*, respectively, which are defined as follows:
  $$\begin{gathered} \partial c(x) = \bigl\{ \xi\in H : c(z) \geq c(x)+ \langle\xi,z-x \rangle, \forall z\in H \bigr\} , \\ \partial q(y) = \bigl\{ \eta\in K : q(u) \geq q(y)+ \langle\eta,u-y \rangle, \forall u\in K \bigr\} .\end{gathered} $$

We note that in (H1), the differentiability of *c* and *q* are not assumed. The representations of *C* and *Q* in (H1) are therefore general enough, because any system of inequalities $c_{i}(x) \leq0$, $i \in I$ and any system of inequalities $q_{j} (y) \leq0$, $j\in J$, where $c_{i}$, $q_{j}$ are convex (not necessarily differentiable) functions, and *I*, *J* are two arbitrary index sets, can be reformulated as the single inequality $c(x) \leq0$ and the single inequality $q(y) \leq0$ with $c(x) = \sup\{c_{i}(x):i\in I\}$ and $q(y) = \sup\{q_{j}(y):j\in J\}$, respectively. Moreover, every convex functions defined on a finite-dimensional Hilbert space is subdifferentiable and its subdifferential operator is a bounded operator on any bounded subset in Hilbert space (see [29]).

### Theorem 4.1

*Let* *C* *and* *Q* *be two nonempty closed convex subsets of real Hilbert spaces* *H* *and* *K*, *respectively*, *satisfy the conditions* (H1) *and* (H2). *Let* $A: H \rightarrow K$ *be a bounded linear operator and* $h:H\rightarrow H$ *be an* *α*-*contraction mapping*. *Assume that the SFP* (1.1) *has a nonempty solution set* Γ *and let* $\{ x_{n}\} \subset H$ *be a sequence generated by*

$$ \textstyle\begin{cases} x_{0} \in H, \\ C_{n} = \{x\in H : c(x_{n})+ \langle\xi_{n},x-x_{n} \rangle\leq 0 \},\\ Q_{n} = \{y\in K : q(Ax_{n})+ \langle\eta_{n},y-Ax_{n} \rangle \leq0 \}, \\ x_{n+1} = \alpha_{n} x_{n}+\beta_{n} h(x_{n}) +\gamma_{n} P_{C_{n}}(x_{n}- \lambda _{n} \nabla g_{\epsilon_{n}}(x_{n}) ), \quad\forall n=0,1,2,\ldots, \end{cases} $$

*where* $\xi_{n} \in\partial c(x_{n})$, $\eta_{n} \in\partial q(Ax_{n})$, $\alpha _{n}+\beta_{n}+\gamma_{n} = 1$ *and*

$$\begin{gathered} g_{\epsilon_{n}}(x_{n}) = \frac{1}{2} \bigl\Vert (I-P_{Q_{n}})Ax_{n} \bigr\Vert ^{2}+ \frac {\epsilon_{n}}{2} \Vert x_{n} \Vert ^{2}, \\ \nabla g_{\epsilon_{n}}(x_{n}) = A^{*} (I-P_{Q_{n}})Ax_{n}+ \epsilon_{n} x_{n} \neq 0 ,\qquad \lambda_{n} = \frac{\rho_{n} g_{\epsilon_{n}}(x_{n})}{ \Vert \nabla g_{\epsilon_{n}}(x_{n}) \Vert ^{2} + \Vert \nabla g_{\epsilon_{n}}(x_{n}) \Vert +\rho_{n} g_{\epsilon_{n}}(x_{n})}\end{gathered} $$

*for all* $n=0,1,2,\ldots$ . *If the sequences* $\{\rho_{n} \}\subset[a,b]$ *for some* $a,b \in(0,2)$, $\{ \alpha_{n} \} \subset[c,d]$ *for some* $c,d \in (0,1)$ *and* $\{\beta_{n} \},\{\gamma_{n} \}\subset(0,1)$ *satisfy the following conditions*:

(i) $0 \leq\epsilon_{n} \leq\beta_{n}^{2m}$ *such that* $m>1$ *for all* $n=0,1,2,\ldots $ ,
(ii) $\lim_{n\rightarrow\infty}\beta_{n} = 0$ *and* $\sum_{n=0}^{\infty}\beta_{n} = \infty$,

*then the sequence* $\{x_{n}\}$ *converges strongly to* $x^{*} \in\Gamma$ *where* $x^{*} = P_{\Gamma}h(x^{*})$.

### Proof

Fix $n\in\mathbb{N}\cup\{0\}$. Observe that the halfspaces $C_{n}$ and $Q_{n}$ are closed and convex sets, and contain *C* and *Q*, respectively. Pick $p \in\Gamma$. We have $p\in C \subset C_{n}$ and $Ap\in Q \subset Q_{n}$. Taking $P_{C_{n}}$ and $P_{Q_{n}}$ in place of *S* and *T*, respectively, in similar arguments to that of the proof in Theorem 3.1, we have $\{x_{n}\}$ is bounded, and by similar arguments, there exists a unique element $x^{*}\in H$ such that $x^{*} = P_{\Gamma}h(x^{*})$. That is $x^{*} \in\Gamma$. We consider into two cases.

Case 1. Assume that $\{\|x_{n}-p\|\}$ is a monotone sequence. By similar arguments, there exists a subsequence $\{x_{n_{k}}\}$ of $\{x_{n}\}$ which converges weakly to $w \in H$, and we have

$$\lim_{k\rightarrow\infty} \bigl\Vert (I-P_{Q_{n_{k}}})Ax_{n_{k}} \bigr\Vert = 0,\qquad \lim_{k\rightarrow\infty} \bigl\Vert (I-P_{C_{n_{k}}})x_{n_{k}} \bigr\Vert = 0. $$

By the definitions of $C_{n_{k}}$ and $Q_{n_{k}}$ we have

$$c(x_{n_{k}}) \leq \langle\xi_{n_{k}},x_{n_{k}}-P_{C_{n_{k}}}x_{n_{k}} \rangle\leq\xi \bigl\Vert (I-P_{C_{n_{k}}})x_{n_{k}} \bigr\Vert $$

and

$$q(Ax_{n_{k}}) \leq \bigl\langle \eta _{n_{k}},Ax_{n_{k}}-P_{Q_{n_{k}}}(Ax_{n_{k}}) \bigr\rangle \leq\eta \bigl\Vert (I-P_{Q_{n_{k}}})Ax_{n_{k}} \bigr\Vert , $$

where $\|\xi_{n}\| \leq\xi< \infty$ and $\|\eta_{n}\| \leq\eta< \infty$ for all $n = 0,1,2,\ldots $ . Hence,

$$\lim_{k\rightarrow\infty} c(x_{n_{k}}) \leq0,\qquad \lim _{k\rightarrow \infty} q(Ax_{n_{k}}) \leq0. $$

Therefore, by the w-lsc of *c* and *q* at *w* and *Aw*, respectively, applying Lemma 2.10, we have

$$c(w) \leq\liminf_{k\rightarrow\infty}c(x_{n_{k}}) \leq0, \qquad q(Aw) \leq \liminf_{k\rightarrow\infty}q(Ax_{n_{k}}) \leq0. $$

It follows that $w \in C$ and $Aw \in Q$. That is, $w\in\Gamma$. By similar arguments, we have the sequence $\{x_{n}\}$ converges strongly to $x^{*} = P_{\Gamma}h(x^{*})$.

Case 2. Assume that $\{\|x_{n}-p\|\}$ is not a monotone sequence. Following similar arguments to those in Case 1 and Case 2 of the proof in Theorem 3.1, we have $\omega_{w}(x_{\tau(n)}) \subset \Gamma$. By similar arguments, we have the sequence $\{x_{n}\}$ converging strongly to $x^{*} = P_{\Gamma}h(x^{*})$. This completes the proof. □

### Corollary 4.2

*Let* *C* *and* *Q* *be two nonempty closed convex subsets of real Hilbert spaces* *H* *and* *K*, *respectively*, *satisfy the conditions* (H1) *and* (H2). *Let* $A: H \rightarrow K$ *be a bounded linear operator and* $h:H\rightarrow H$ *be an* *α*-*contraction mapping*. *Assume that the SFP* (1.1) *has a nonempty solution set* Γ *and let* $\{ x_{n}\} \subset H$ *be a sequence generated by*

$$ \textstyle\begin{cases} x_{0} \in H, \\ C_{n} = \{x\in H : c(x_{n})+ \langle\xi_{n},x-x_{n} \rangle\leq 0 \},\\ Q_{n} = \{y\in K : q(Ax_{n})+ \langle\eta_{n},y-Ax_{n} \rangle \leq0 \}, \\ x_{n+1} = \alpha_{n} x_{n}+\beta_{n} h(x_{n}) +\gamma_{n} P_{C_{n}}(x_{n}- \lambda _{n} \nabla g(x_{n}) ), \quad\forall n=0,1,2,\ldots, \end{cases} $$

*where* $\xi_{n} \in\partial c(x_{n})$, $\eta_{n} \in\partial q(Ax_{n})$, $\alpha _{n}+\beta_{n}+\gamma_{n} = 1$ *and* $g(x_{n}) = \frac{1}{2}\|(I-P_{Q_{n}})Ax_{n}\|^{2}$,

$$\nabla g(x_{n}) = A^{*} (I-P_{Q_{n}})Ax_{n}\neq0,\qquad \lambda_{n} = \frac{\rho_{n} g(x_{n})}{ \Vert \nabla g(x_{n}) \Vert ^{2} + \Vert \nabla g(x_{n}) \Vert +\rho_{n} g(x_{n})} $$

*for all* $n=0,1,2,\ldots$ . *If the sequences* $\{\rho_{n} \}\subset[a,b]$ *for some* $a,b \in(0,2)$, $\{ \alpha_{n} \} \subset[c,d]$ *for some* $c,d \in (0,1)$ *and* $\{\beta_{n} \},\{\gamma_{n} \}\subset(0,1)$ *satisfy the following conditions*: $\lim_{n\rightarrow\infty}\beta_{n} = 0$ *and* $\sum_{n=0}^{\infty}\beta_{n} = \infty$, *then the sequence* $\{x_{n}\}$ *converges strongly to* $x^{*} \in\Gamma$ *where* $x^{*} = P_{\Gamma}h(x^{*})$.

## Numerical results

In this section, we give some insight into the behavior of the algorithms presented in Corollary 3.4 and Corollary 4.2. We implemented them in Mathematica to solve and run on a computer Pentium(R) mobile processor 1.50 GHz. We use $\|x_{n+1}-x_{n}\| _{2} < \epsilon$ as the stopping criterion.

Throughout the computational experiments, the parameters used in those algorithms were sets as $\epsilon= 10^{-6}$, $\rho_{n} = 1$, $\alpha_{n} = \frac{1}{2}$, $\beta_{n} = \frac{1}{n+3}$ and $\gamma_{n} = 1-\alpha_{n} -\beta_{n}$ for all $n=0,1,2,\ldots$ . In the results report below, all CPU times reported are in seconds. The approximate solution is referred to the last iteration.

In the computation, the projection $P_{C}$ where *C* is a closed ball in $\mathbb{R}^{N}$, and in the projections $P_{C_{n}}$ and $P_{Q_{n}}$ where $C_{n}$ and $Q_{n}$ are the halfspaces in $\mathbb{R}^{N}$ and $\mathbb {R}^{M}$, respectively, we use the formulation as follows.

### Proposition 5.1

*For* $\rho>0$ *and* $C = \{x\in\mathbb{R}^{N}:\|x\|_{2}\leq\rho\}$, *we have*

$$P_{C} x = \textstyle\begin{cases} \frac{\rho x}{ \Vert x \Vert _{2}} ,& x \notin C; \\ x ,& x \in C. \end{cases} $$

### Proposition 5.2

([30, 31])

*Let* $H=(\mathbb{R}^{N},\|\cdot\| _{2})$ *and* $K=(\mathbb{R}^{M},\|\cdot\|_{2})$. *Assume that* *C* *and* *Q* *satisfy the conditions* (H1) *and* (H2), *and define the halfspaces* $C_{n}$ *and* $Q_{n}$ *as algorithm in Corollary* 4.2. *For any* $z\in \mathbb{R}^{N}$ *and for each* $n=0,1,2,\ldots $ *we have*

$$P_{C_{n}}(z) = \textstyle\begin{cases} z-\frac{c(x_{n})+ \langle\xi_{n},z-x_{n} \rangle}{ \Vert \xi_{n} \Vert ^{2}}\xi_{n} ,& c(x_{n})+ \langle\xi_{n},z-x_{n} \rangle> 0; \\ z ,& c(x_{n})+ \langle\xi_{n},z-x_{n} \rangle\leq0, \end{cases} $$

*and*

$$P_{Q_{n}}(Az) = \textstyle\begin{cases} Az-\frac{q(Ax_{n})+ \langle\eta_{n},Az-Ax_{n} \rangle}{ \Vert \eta _{n} \Vert ^{2}}\eta_{n} ,& q(Ax_{n})+ \langle\eta_{n},Az-Ax_{n} \rangle > 0; \\ Az ,& q(Ax_{n})+ \langle\eta_{n},Az-Ax_{n} \rangle\leq0. \end{cases} $$

### Example 5.3

(A projection point problem)

Let $C=\{a\in \mathbb{R}^{4}:\|a\|_{2}\leq3\}$ and $u\in\mathbb{R}^{4}$ chosen arbitrarily. Find a unique solution $x^{*} \in C$ which is nearest the point *u* and satisfies the following system of linear equations:

$$\textstyle\begin{cases} x+2y+3z+w = 1, \\ x-y+z-2w = 2, \\ x+y-2z+w = 3, \end{cases} $$

where $x,y,z,w \in\mathbb{R}$.

Let $H=(\mathbb{R}^{4},\|\cdot\|_{2})$ and $K=(\mathbb{R}^{3},\|\cdot\| _{2})$. Take

$$ A= \begin{pmatrix} 1 & 2 & 3 & 1 \\ 1 & -1 & 1 & -2 \\ 1 & 1 & -2 & 1 \end{pmatrix} ,\quad\quad Q = \bigl\{ b:b=(1,2,3)^{T} \bigr\} $$

and

$$h(x) = u $$

for all $x\in\mathbb{R}^{4}$ into Corollary 3.4, we have

$$\textstyle\begin{cases} x_{0} \in\mathbb{R}^{4} \quad\text{chosen arbitrarily}, \\ x_{n+1}=\alpha_{n} x_{n} +\beta_{n} u+\gamma_{n} P_{C} (x_{n}-\lambda _{n}(A^{*} Ax_{n}-A^{*} b) ), \quad\forall n=0,1,2,\ldots, \end{cases} $$

where $b=(1,2,3)^{T}$, $\lambda_{n} = \frac{\| Ax_{n}-b \|^{2}}{2\| A^{*}Ax_{n} -A^{*}b \|^{2}+2\| A^{*}Ax_{n} -A^{*}b \|+\| Ax_{n}-b \|^{2}}$ if $Ax_{n} \neq b$ and $\lambda_{n} = 0$ if $Ax_{n} = b$ for all $n=0,1,2,\ldots$ . As $n\rightarrow \infty$, we have $x_{n} \rightarrow x^{*}$ such that $x^{*}$ is the our solution, which depends on the point *u* and $x_{0}$. The numerical results are listed in Table 1 using the different points *u* and the different starting points $x_{0}$.

**Table 1:** Results for Example 5.3 using the algorithm in Corollary 3.4

Choiceness *u*	Starting points $x_{0}$	Number of iterations	CPU (s)	Approximate solution $x^{*}$ (approximates of $Ax^{*}$ and $\|x^{*}\|_{2}$)
$(0,0,0,0)^{T}$	$(0,0,0,0)^{T}$	9500	5.317	$(1.974662,0.512779,-0.498849,-0.508389)^{T}$
$(0,0,0,0)^{T}$	$(1,1,1,1)^{T}$	9501	5.187	$(1.974695,0.512718,-0.498837,-0.508336)^{T}$
$(0,0,0,0)^{T}$	$(1,2,3,4)^{T}$	9505	5.217	$(1.974803,0.512523,-0.498798,-0.508168)^{T}$
				$Ax^{*} = (0.99528,1.97981,2.97675)^{T}$, $\|x^{*}\|_{2} = 2.16091$
$(1,1,1,1)^{T}$	$(0,0,0,0)^{T}$	9406	5.488	$(2.130245,0.226710,-0.439453,-0.255758)^{T}$
$(1,1,1,1)^{T}$	$(1,1,1,1)^{T}$	9406	5.888	$(2.130278,0.226649,-0.439440,-0.255705)^{T}$
$(1,1,1,1)^{T}$	$(1,2,3,4)^{T}$	9409	5.568	$(2.130386,0.226451,-0.439402,-0.255534)^{T}$
				$Ax^{*} = (1.00955,1.97560,2.98010)^{T}$, $\|x^{*}\|_{2} = 2.20179$
$(1,2,3,4)^{T}$	$(0,0,0,0)^{T}$	12,180	4.977	$(2.641308,-0.734424,-0.244857,0.583179)^{T}$
$(1,2,3,4)^{T}$	$(1,1,1,1)^{T}$	12,179	5.127	$(2.641332,-0.734471,-0.244847,0.583221)^{T}$
$(1,2,3,4)^{T}$	$(1,2,3,4)^{T}$	12,178	5.387	$(2.641411,-0.734620,-0.244817,0.583350)^{T}$
				$Ax^{*} = (1.02107,1.96452,2.97978)^{T}$, $\|x^{*}\|_{2} = 2.81353$

### Example 5.4

(A split feasibility problem)

Let

$$ A= \begin{pmatrix} 2 & -1 & 3 \\ 4 & 2 & 5 \\ 2 & 0 & 2 \end{pmatrix} ,\qquad C = \bigl\{ (x,y,z)\in\mathbb{R}^{3}:x+y^{2}+2z \leq0 \bigr\} $$

and

$$Q= \bigl\{ (x,y,z)\in\mathbb{R}^{3} : x^{2}+y-z \leq0 \bigr\} . $$

Find some point $x^{*}\in C$ with $Ax^{*} \in Q$.

Let $H=(\mathbb{R}^{3},\|\cdot\|_{2})$ and $K=(\mathbb{R}^{3},\|\cdot\| _{2})$. Take $c(x,y,z) = x+y^{2}+2z$, $q(x,y,z) = x^{2}+y-z$ and $h(x,y,z)=0$ for all $x,y,z\in\mathbb{R}$ into Corollary 4.2, we have

$$\textstyle\begin{cases} x_{0} \in\mathbb{R}^{3} \quad\text{chosen arbitrarily}, \\ x_{n+1}=\alpha_{n} x_{n} +\gamma_{n} P_{C_{n}} (x_{n}-\lambda_{n}(A^{*} Ax_{n}-A^{*} P_{Q_{n}}(Ax_{n})) ), \quad\forall n=0,1,2,\ldots, \end{cases} $$

where $\lambda_{n} = \frac{\| Ax_{n}-P_{Q_{n}}(Ax_{n}) \|^{2}}{2\| A^{*}Ax_{n} -A^{*} P_{Q_{n}}(Ax_{n}) \|^{2}+2\| A^{*}Ax_{n} -A^{*} P_{Q_{n}}(Ax_{n}) \|+\| Ax_{n}-P_{Q_{n}}(Ax_{n}) \|^{2}}$ if $Ax_{n} \notin Q_{n}$ and $\lambda_{n} = 0$ if $Ax_{n} \in Q_{n}$ for all $n=0,1,2,\ldots$ . As $n\rightarrow\infty$, we have $x_{n} \rightarrow x^{*}$ such that $x^{*}$ is the our solution, which depends on the zero point and $x_{0}$. The numerical results are listed in Table 5 using the different starting points $x_{0}$, and we compare the results of Qu and Xiu [6, 7], and Li [8], which are listed in Table 2, Table 3 and Table 4, respectively. We found that in the calculation approximate value of $q(Ax^{*})$ using the our algorithm method was fit to the solution than the algorithms method of Qu and Xiu, and Li.

**Table 2:** Results for Example 5.4 using Qu and Xiu method in [7]

Starting points $x_{0}$	Number of iterations	CPU (s)	Approximate solution $x^{*}$	Approximate of $c(x^{*})$	Approximate of $q(Ax^{*})$
$(1,2,3,0,0,0)^{T}$	1890	2.7740	$(-0.1203,0.0285,0.0582)^{T}$	−0.00308775	−0.0152279
$(1,1,1,1,1,1)^{T}$	2978	4.2860	$(0.8603,-0.1658,-0.5073)^{T}$	−0.12681	1.08162
$(1,2,3,4,5,6)^{T}$	3317	4.8570	$(3.6522,-0.1526,-2.3719)^{T}$	−1.06831	15.5579

**Table 3:** Results for Example 5.4 using Qu and Xiu method in [6]

Starting points $x_{0}$	Number of iterations	CPU (s)	Approximate solution $x^{*}$	Approximate of $c(x^{*})$	Approximate of $q(Ax^{*})$
$(1,2,3)^{T}$	64	0.1570	$(-0.4019,0.0674,0.1967)^{T}$	−0.00395724	0.0322236
$(1,1,1)^{T}$	81	0.0940	$(0.3568,0.0343,-0.2652)^{T}$	−0.172424	0.426806
rand(3,1)∗10	105	0.0940	$(0.8747,0.0795,-0.6876)^{T}$	−0.49418	1.5322

**Table 4:** Results for Example 5.4 using Li method in Algorithm 1 [8]

Starting points $x_{0}$	Number of iterations	CPU (s)	Approximate solution $x^{*}$	Approximate of $c(x^{*})$	Approximate of $q(Ax^{*})$
$(1,2,3)^{T}$	4	0.1410	$(-0.4024,0.0658,0.1958)^{T}$	−0.00647036	0.0319258
$(1,1,1)^{T}$	5	0.0940	$(0.3532,0.0392,-0.2707)^{T}$	−0.186663	0.43465
rand(3,1)∗10	8	0.0940	$(0.8768,0.0604,-0.6844)^{T}$	−0.488352	1.51358

**Table 5:** Results for Example 5.4 using Algorithm in Corollary 4.2

Starting points $x_{0}$	Number of iterations	CPU (s)	Approximate solution $x^{*}$	Approximate of $c(x^{*})$	Approximate of $q(Ax^{*})$
$(1,2,3)^{T}$	1220	1.492	$(-0.0009,0.0007,0.0002)^{T}$	−0.00049951	0.00050081
$(1,1,1)^{T}$	1062	1.342	$(0.0004,0.0007,-0.0006)^{T}$	−0.00079951	0.00130016
$(4,5,6)^{T}$	1225	1.642	$(0.0006,0.0007,-0.0007)^{T}$	−0.00079951	0.00140036
rand(3,1)∗10	1197	1.832	$(0.0008,0.0004,-0.0007)^{T}$	−0.00059984	0.00110064
$(6,5,4)^{T}$	2569	4.346	$(0.0020,0.0004,-0.0015)^{T}$	−0.00099984	0.00190400
$(2,2,2)^{T}$	1365	2.083	$(0.0007,0.0006,-0.0008)^{T}$	−0.00089964	0.00140049
$(3,2,1)^{T}$	2093	3.525	$(0.0015,0.0005,-0.0013)^{T}$	−0.00109975	0.00180225

### Example 5.5

(A convex feasibility problem) Let $C=\{a\in \mathbb{R}^{3} : 2 \leq\|a\|_{2} \leq3 \}$. Find some point $x^{*} \in C$ which satisfies the following system of nonlinear inequalities:

$$\textstyle\begin{cases} y^{2}+z^{2}-4 \leq0, \\ -x^{2}+z-1 \leq0, \end{cases} $$

where $x,y,z\in\mathbb{R}$.

Let $H=(\mathbb{R}^{3},\|\cdot\|_{2})$, $K=(\mathbb{R}^{3},\|\cdot\|_{2})$ and $u\in C$. Take $A=I$, $h(x,y,z)=u$ and

$$\begin{gathered} C= \bigl\{ (x,y,z)\in\mathbb{R}^{3}:c(x,y,z)=\sup \bigl\{ c_{1}(x,y,z),c_{2}(x,y,z) \bigr\} \leq0 \bigr\} , \\ Q= \bigl\{ (x,y,z)\in\mathbb{R}^{3} : q(x,y,z)=\sup \bigl\{ q_{1}(x,y,z),q_{2}(x,y,z) \bigr\} \leq0 \bigr\} ,\end{gathered} $$

such that $c_{1}(x,y,z)=x^{2}+y^{2}+z^{2}-9$, $c_{2}(x,y,z)=-x^{2}-y^{2}-z^{2}+4$, $q_{1}(x,y,z)=y^{2}+z^{2}-4$ and $q_{2}(x,y,z)=-x^{2}+z-1$ for all $x,y,z\in \mathbb{R}$ into Corollary 4.2, we have

$$\textstyle\begin{cases} x_{0} \in\mathbb{R}^{3} \quad\text{chosen arbitrarily}, \\ x_{n+1}=\alpha_{n} x_{n} +\beta_{n} u+\gamma_{n} P_{C_{n}} (x_{n}-\lambda _{n}(x_{n}-P_{Q_{n}}x_{n}) ), \quad\forall n=0,1,2,\ldots, \end{cases} $$

where $\lambda_{n} = \frac{\| x_{n}-P_{Q_{n}}x_{n} \|}{3\| x_{n} -P_{Q_{n}}x_{n} \| +2}$ for all $n=0,1,2,\ldots$ . As $n\rightarrow\infty$, we have $x_{n} \rightarrow x^{*}$ such that $x^{*}$ is the our solution, which depends on the point *u* and $x_{0}$. The numerical results are listed in Table 6 using the different points $u\in C$ and the different starting points $x_{0}$.

**Table 6:** Results for Example 5.5 using Algorithm in Corollary 4.2

Choiceness *u*	Starting points $x_{0}$	Number of iterations	CPU (s)	Approximate solution $x^{*}$	Approximate of $q_{1}(x^{*})$	Approximate of $q_{2}(x^{*})$	Approximate of $\|x^{*}\|_{2}$
$(2,0,0)^{T}$	$(1,1,1)^{T}$	1901	3.295	$(1.999999,0.001343,0.001343)^{T}$	−4.00000	−4.99865	2.00000
$(2,0,0)^{T}$	$(2,2,2)^{T}$	2336	4.486	$(1.999998,0.001651,0.001651)^{T}$	−3.99999	−4.99834	2.00000
$(2,0,0)^{T}$	$(1,2,3)^{T}$	2716	4.576	$(1.999998,0.001506,0.002259)^{T}$	−3.99999	−4.99773	2.00000
$(3,0,0)^{T}$	$(1,1,1)^{T}$	2209	3.935	$(2.998244,0.000948,0.000948)^{T}$	−4.00000	−9.98852	2.99824
$(3,0,0)^{T}$	$(2,2,2)^{T}$	2417	3.505	$(2.999133,0.001595,0.001595)^{T}$	−3.99999	−9.99320	2.99913
$(3,0,0)^{T}$	$(1,2,3)^{T}$	2821	3.955	$(2.998563,0.001346,0.002019)^{T}$	−3.99999	−9.98936	2.99856
$(1,2,0)^{T}$	$(1,1,1)^{T}$	1667	3.375	$(1.000131,1.998964,0.001299)^{T}$	−0.00414	−1.99896	2.23520
$(1,2,0)^{T}$	$(2,2,2)^{T}$	2068	3.745	$(1.000919,1.999901,0.001849)^{T}$	−0.00039	−1.99999	2.23639
$(1,2,0)^{T}$	$(1,2,3)^{T}$	2363	4.566	$(0.999977,1.999833,0.002357)^{T}$	−0.00066	−1.99760	2.23591

### Example 5.6

(A convex minimization problem)

Find minimize of $f(x,y,z)=(x-2)^{2}+ (y-2)^{2}+(z-3)^{2}$ subject to the constraint $g(x,y,z)=x^{2}+y^{2}+z^{2}-4 = 0$ where $x,y,z \in\mathbb{R}$.

Define the Lagrange function $L(x,y,z,\lambda)$ as follows:

$$\begin{aligned} L(x,y,z,\lambda) &= f(x,y,z)+\lambda g(x,y,z) \\ &= (x-2)^{2}+(y-2)^{2}+(z-3)^{2} +\lambda \bigl(x^{2}+y^{2}+z^{2}-4 \bigr),\end{aligned} $$

where $x,y,z,\lambda\in\mathbb{R}$. Hence, the our solution set is equivalent to the solution set of the following system of nonlinear equations:

$$\textstyle\begin{cases} 2(x-2)+2\lambda x = L_{x} = 0, \\ 2(y-2)+2\lambda y = L_{y} = 0, \\ 2(z-3)+2\lambda z =L_{z} = 0, \\ x^{2}+y^{2}+z^{2}-4 = L_{\lambda}= 0. \end{cases} $$

Let $H=(\mathbb{R}^{4},\|\cdot\|_{2})$ and $K=(\mathbb{R}^{4},\|\cdot\| _{2})$. Take $A=I$, $h(x,y,z,\lambda)=0$ and

$$\begin{gathered} C= \Bigl\{ (x,y,z,\lambda)\in\mathbb{R}^{4}:c(x,y,z,\lambda)=\sup _{1\leq i \leq4} -q_{i}(x,y,z,\lambda) \leq0 \Bigr\} , \\ Q= \Bigl\{ (x,y,z,\lambda)\in\mathbb{R}^{4}:q(x,y,z,\lambda)=\sup _{1\leq i \leq4} q_{i}(x,y,z,\lambda) \leq0 \Bigr\} ,\end{gathered} $$

such that

$$\begin{gathered} q_{1}(x,y,z,\lambda)=2(x-2)+2\lambda x,\qquad q_{2}(x,y,z,\lambda )=2(y-2)+2\lambda y, \\ q_{3}(x,y,z,\lambda)=2(z-3)+2\lambda z, \qquad q_{4}(x,y,z, \lambda)=x^{2}+y^{2}+z^{2}-4,\end{gathered} $$

for all $x,y,z,\lambda\in\mathbb{R}$ into Corollary 4.2, we have

$$\textstyle\begin{cases} x_{0} \in\mathbb{R}^{4} \quad\text{chosen arbitrarily}, \\ x_{n+1}=\alpha_{n} x_{n} +\gamma_{n} P_{C_{n}} ( x_{n} - \lambda_{n} (x_{n}-P_{Q_{n}}x_{n}) ) , \quad\forall n=0,1,2,\ldots, \end{cases} $$

where $\lambda_{n} = \frac{\| x_{n}-P_{Q_{n}}x_{n} \|}{3\| x_{n} -P_{Q_{n}}x_{n} \| +2}$ for all $n=0,1,2,\ldots$ . As $n\rightarrow\infty$, we have $x_{n} \rightarrow x^{*}$ such that the our solution is $\frac{2}{\sqrt {17}}(2,2,3)^{T}$ with some Lagrange multiplier *λ*, which depends on the zero point and $x_{0}$. The numerical results are listed in Table 7 using the different starting points $x_{0}$, and we switch the stopping criterion *ϵ* to 10^−4^ for the verification to the our solution.

**Table 7:** Results for Example 5.6 using Algorithm in Corollary 4.2

Starting points $x_{0}$	Number of iterations	CPU (s)	Approximate solution	Lagrange multiplier
$(1,2,1,0)^{T}$	15,562	17.745	$(0.969812,0.969905,1.455039)^{T}$	1.061319
$(2,2,2,0)^{T}$	15,566	14.511	$(0.969905,0.969812,1.455039)^{T}$	1.061319
$(1,2,3,0)^{T}$	15,567	11.666	$(0.969812,0.969905,1.455039)^{T}$	1.061319
$(4,5,6,0)^{T}$	15,566	17.495	$(0.969812,0.969905,1.455039)^{T}$	1.061319

## Conclusion

In this paper, we obtain an iterative scheme using the gradient projection method with a new step size, which is not depend on the related matrix inverses and the largest eigenvalue (or the spectral radius of the self-adjoint operator) of the related matrix, based on Moudafi’s viscosity approximation method for solving the *split common fixed point problem* (SCFP) for two firmly nonexpansive mappings and also solving the *split feasibility problem* (SFP) such that other strong convergence theorems for the SCFP and the SFP are obtained.
